# Synthetic Perturbations in IL6 Biological Circuit Induces Dynamical Cellular Response

**DOI:** 10.3390/molecules27010124

**Published:** 2021-12-26

**Authors:** Bhavnita Soni, Shailza Singh

**Affiliations:** National Centre for Cell Science, NCCS Complex, Ganeshkhind, SPPU Campus, Pune 411007, India; bhavnitas@nccs.res.in

**Keywords:** IL6, SOCS1, SOCS3, systems biology, synthetic biology, leishmaniasis

## Abstract

Macrophage phenotype plays a crucial role in the pathogenesis of Leishmanial infection. Pro-inflammatory cytokines signals through the Janus kinase/signal transducer and activator of transcription (JAK/STAT) pathway that functions in parasite killing. Suppression of cytokine signaling (SOCS) is a well-known negative feedback regulator of the JAK/STAT pathway. However, change in the expression levels of SOCSs in correlation with the establishment of infection is not well understood. IL6 is a pleotropic cytokine that induces SOCS1 and SOCS3 expression through JAK-STAT signaling. Mathematical modeling of the TLR2 and IL6 signaling pathway has established the immune axis of SOCS1 and SOCS3 functioning in macrophage polarization during the early stage of Leishmania major infection. The ratio has been quantified both in silico and in vitro as 3:2 which is required to establish infection during the early stage. Furthermore, phosphorylated STAT1 and STAT3 have been established as an immunological cross talk between TLR2 and IL6 signaling pathways. Using synthetic biology approaches, peptide based immuno-regulatory circuits have been designed to target the activity of SOCS1 which can restore pro-inflammatory cytokine expression during infection. In a nutshell, we explored the potential of synthetic biology to address and rewire the immune response from Th2 to Th1 type during the early stage of leishmanial infection governed by SOCS1/SOCS3 immune axis.

## 1. Introduction

Leishmaniasis is the second most neglected tropical disease after malaria, and it is estimated that between 600,000 to 1 million new cases of cutaneous leishmaniasis occur worldwide annually [1]. Cutaneous leishmaniasis (CL) is an immune mediated skin pathology characterized by crusted papules or ulcers on exposed skin [2]. The causative agents are *Leishmania major*, *L. tropica*, *L. braziliensis*, *L. mexicana*, and *L. amazonensis* [2]. The immunobiology of CL includes the initial interaction of parasite’s lipophosphoglycan (LPG) to TLR2 of host macrophage which results in initiation of two types of responses [3]. First, Th1 response is characterized by higher production of pro-inflammatory cytokines such as IL12, TNFα, IL1β and IFNγ [4]. IFNγ classically activates the macrophages resulting in activation of ROS dependent pathway for killing of parasites inside the macrophage and the phenotype is called M1 phenotype [4]. Second is the Th2 response which is characterized by anti-inflammatory cytokines production such as IL4, IL10, TGFβ, IL6, etc. IL4 and IL10 cytokines alternatively activate the macrophages in such a way that the parasite survives inside the macrophages and the resultant phenotype is called M2 phenotype [5]. The outcome of the infection or the disease manifestation depends upon the balance between Th1/Th2 response [6,7]. 

IL6 is pleiotropic in nature, and functions as both a pro-inflammatory as well as an anti-inflammatory cytokine [8]. IL6 functions through JAK-STAT1 signaling, causing production of excess pro- inflammatory cytokines such as TNF α, IL1β and thus shifting the macrophage polarization towards M1 phenotype [9]. Whereas, through JAK-STAT3 signaling, the expression of anti-inflammatory cytokines is induced such as IL4 and IL10 converting the macrophage into M2 phenotype [5,10]. Suppressor of cytokine signaling (SOCS) is a negative feedback regulator of JAK-STAT signaling pathway; it binds to gp130 and inhibits the phosphorylation of STATs, thereby inhibiting the JAK-STAT pathway. If the ratio of SOCS1/SOCS3 is increased, then JAK STAT1 signaling shuts down, shifting the macrophage phenotype towards M2 phenotype and apparently if the ratio is decreased, JAK-STAT3 signaling pathway shuts down, thereby shifting the macrophage towards M1 phenotype [10,11,12].

Suppressor of cytokine signaling (SOCS) isoforms plays a crucial role in pathogenesis of many infectious diseases. SOCS1 is a crucial host factor that regulates the intracellular dynamism of HIV-1 Gag during HIV infection [13]. It is also found to regulate macrophage polarization during infection of Brugia malayi, a nematode, one of the three causative agents of lymphatic filariasis in humans [12]. Furthermore, SOCS1 has also been shown to be an influenza virus-induced virulence factor that enhances the infection of cells; therefore it has been targeted through the antagonist to treat infection in C57BL/6 mice [14]. In leishmaniasis, SOCS isoforms play a crucial role in the establishment of infection [15]. 

We all know that the immune system is a tremendously complex system which needs to be understood through mathematical models of varied and myriad number of interconnecting components. We have already determined the role of SOCS1/SOCS3 ratio in establishing the immune response during the early stage of L. major infection through mathematical modeling (BIOMD0000000873) [11]. The model was based on experimental data obtained from the literature [16] as well as our predictions, which further depicted the role of TLR2 and IL6 signaling pathways during the early stage of infection. Since in-systems biology predictions and experimentation go hand in hand, our first aim was to validate the ratio of SOCS1/SOCS3 in wet lab conditions and, on the basis of this, refine the model for better understanding of underlying disease mechanisms. 

Modeling and simulations are an integral part of systems biology, where mathematical modeling guides’ in vitro/in vivo experimentation which further aid in model refinement leading to better understanding of complex biological systems. Thus, model refinement is an important step toward unfolding the crucial dynamics of complex biological systems [17]. It would be worthwhile to mention here that performing in silico deterministic simulation has greater advantages than the stochastic one. In a deterministic model, a particular set of parameters and initial concentration always lead to the same type of model prediction, which is described in the form of ordinary differential equations (ODEs) depicting the most appropriate behavior of the system, whereas stochastic simulation is less complex and accounts for the combinatorial explosion of the parameter and initial concentrations, which gives different model predictions defined in terms of a chemical master equation [18]. In corroboration to this, analysis of the network suggests several model modifications in order to better fit available knowledge and the data, which further helped intrigued our experimental hypothesis to be pursued. Thus, in the present work, using experimental observations we have refined our previous models of healthy state model (HSM) and diseased state model (DSM) established during early stage of Leishmania major infection. HSM refers to M1, and DSM refers to M2 phenotype of macrophage, respectively, during infection. 

Based on predictions of the refined models, our next aim is to design therapeutics based on synthetic biology approaches using biological components (promoter, RBS, RNA polymerase, spacer, etc.) that have been rewired to act as a transcriptional pool. Each biological entity represents one transcription unit. Each reaction set in the synthetic circuit is an intermittent one, with an underlying assumption of the synthetic network behaving as a discrete-stochastic process. Evolvability of the synthetic circuit has also been taken into account through stochastic simulation (varying concentration of each species in the system). Extensive work has been done using tunable synthetic circuit in mammalian cell for therapeutic purpose such as the use of gene circuit for sensing and suppressing inflammation [19], treatment of metabolic syndrome [20], for anti- cancerous gene therapy [21] and the use of synthetic gene circuit for immune mediated therapy [22]. Besides these, synthetic mammalian gene circuits have also been used to deliver specific RNA to cells [23]. The success of the mammalian based synthetic gene regulatory circuit in various diseases has drawn our attention and motivated us to generate a potent novel therapeutics in Leishmaniasis. Here, in this paper, we have assimilated different parts of the transcriptional unit (promoter, RBS, RNA polymerase, spacer etc.), pooled from the genetic pool to generate a functionally active synthetic circuit. The design is precise keeping it modular in fashion which ensures the simple and reproducible workability of the circuit for lest it should not be visualized as a Rube Goldberg machine. To the best of our knowledge, this manuscript serves as the first ever report of IL6 based synthetic gene regulatory circuit for treating L. major infection at cellular level.

**Figure 1:**
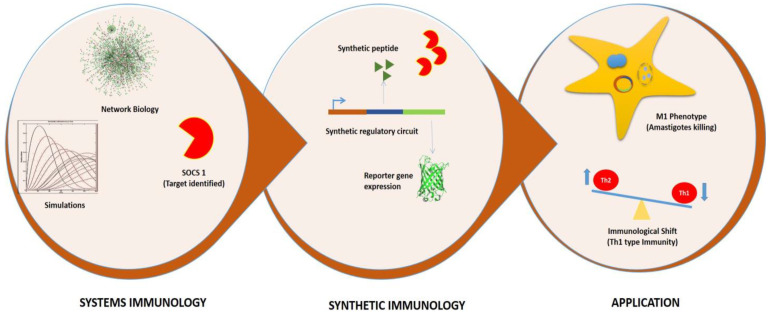
Graphical representation of Systems and Synthetic immunology.

There are various forms of synthetic devices used for therapeutic purposes, more complex types are oscillators and toggle switches which contain two or more stable states with or without intermediate unstable states [24]. The simpler form of a synthetic device is the repressilator, characterized by the presence of a feedback loop with at least three genes out of which one encodes for protein that represses the next gene in loop [25]. In the present study, we have used the combination of toggle switch and repressilator to design the synthetic circuit that may tweak the immune response from Th2 to Th1 type during early stage of leishmanial infection. The immuno based synthetic device serves as the first attempt to revert the anti-inflammatory action of IL6 into its pro-inflammatory behavior through a mathematically established SOCS1/SOCS3 immune axis. Thus, the increasing body of vast knowledge together with comprehensive mathematical analysis may aid immuno-based synthetic devices to become a reality in a Leishmania infection model system (Figure 1). 

## 2. Results

### 2.1. SOCS1/SOCS3 Differential Expression Governing Macrophage Polarization

**Figure 2:**
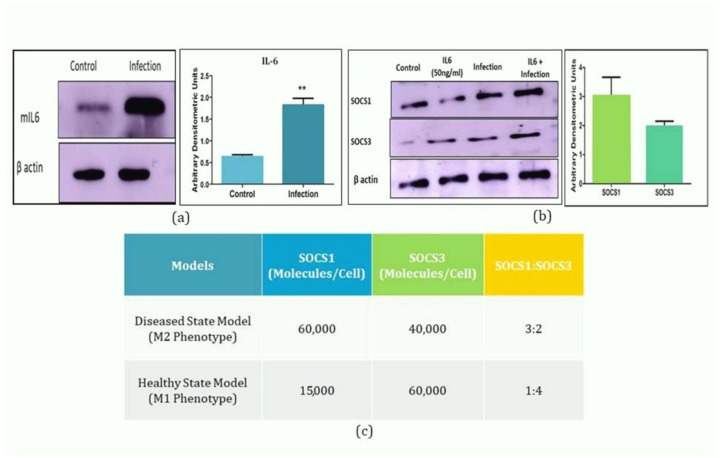
In vitro validation of mathematical model: (**a**) Quantification of IL6 post one hour of infection. (**b**) Quantification of SOCS1/SOCS3 concentration ratio obtained after simulation of mathematical model. (**c**) In silico quantification of SOCS1/SOCS3 ratio. Macrophages were exposed to stationary phase promastigotes in ratio of 1:10 (macrophage: parasite) for 24 h and un-internalized parasite was washed followed by treatment of IL6 (50 ng/mL) for 1 h post infection. Ratio was further quantified through western blotting followed by Densitometric analysis of the blots against b actin level. The experiments were performed thrice. Results from one representative experiment are shown. The densitometric value represents mean ± SD with *p* value ** *p* < 0.01.

Interleukin 6 cytokine is one of the major cytokines which are released during interaction of LPG (Lipophosphoglycan) with TLR2 at an early stage of infection [3]. Previously, we have generated two models deciphering the dual action of IL6 in Leishmaniasis i.e., DSM depicting the anti-inflammatory role and HSM showing pro inflammatory role of IL6 [11]. We have hypothesized that IL6 may act as an anti-inflammatory cytokine causing selective expression of SOCS proteins resulting in converting the macrophage in M2 phenotype during Leishmanial infection. In this process, first the amount of IL6 is quantified during post one hour of L. major infection (Figure 2a) and as per our previous observation, 80 min simulation of the diseased state model shows differential expression of SOCS1 and SOCS3 protein [11]. This crucial finding was further validated in vitro by infecting the macrophages with L. major stationary phase promastigotes in the presence of Interleukin 6 cytokine. Western blot data depicted increased expression of SOCS1 and SOCS3 protein during infection which further got enhanced in presence of IL6 treatment. Densitometric analysis identifies the ratio of SOCS1/SOCS3 as 3:2 post one hour of infection (Figure 2b,c). The obtained data signify the anti-inflammatory role of IL6 in establishing SOCS1/SOCS3 immune axis during the early stage of infection. 

The IL6 mathematical model is constructed for it to be testable through further experimentation. We need to quantify the uncertainty of those predictions, given the information they are built upon. Either the local analysis of sensitivities or the non-local sampling of parameter space can be used to estimate prediction uncertainties. Model re-parameterizations and targeted experiments result in identifiable parameters. Here, in this paper, what we have seen is that even though some parameters in the ensemble vary considerably, the ensemble of trajectories shows much less variation. Nonetheless, the reality is that many mathematical models are published with parameters that do not systematically fit to the data. We would reiterate that our previously published IL6 model (BIOMD0000000873) does not fit the experimental data, leading us to only use the synthetic data generated by the model itself. Counter intuitively, we went ahead in understanding the sensitivity of model predictions to parameters which would suggest possible perturbations of interest and adopted the established protocol of our lab. 

**Figure 3:**
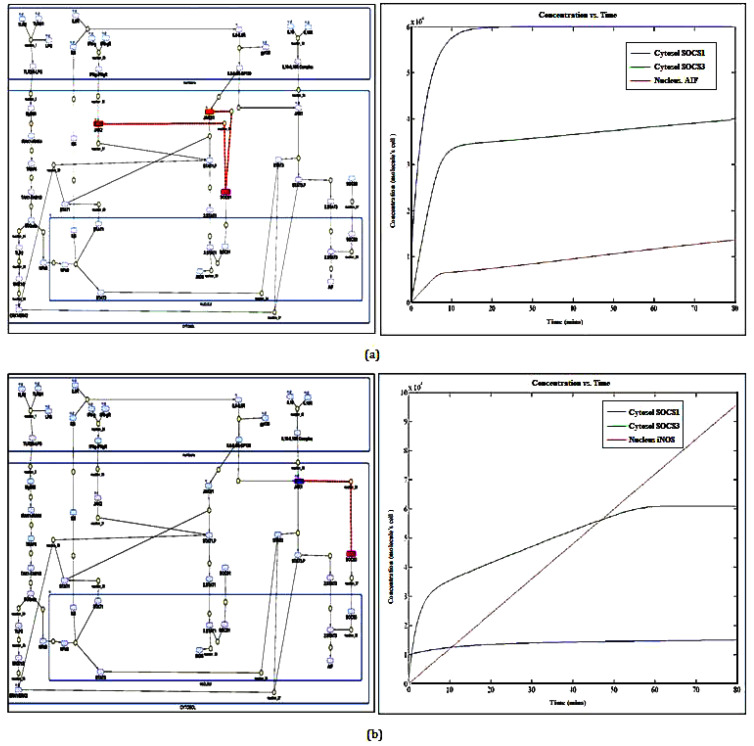
Refinement of TLR2-TLR6 and IL6 signaling pathway: (**a**) Mathematical model of M2 phenotype representing higher production of Anti-inflammatory factors (AIF) and SOCS1, (**b**) Mathematical model of M1 phenotype depicting higher production of iNOS and SOCS3 at the end of 80 min simulation.

On the basis of aforementioned strategies, refinement of the mathematical models i.e., Diseased state (M2 phenotype); DSM and Healthy state (M1 phenotype); HSM have been performed. Each model contained 46 species comprising 41 reactions in DSM and 40 reactions in HSM, respectively (Supplementary Material S1). After a simulation for 80 min, DSM shows increased concentration of Anti-inflammatory factors (AIF) such as IL10, TGFb, etc., as well as SOCS1 protein whereas HSM shows higher concentration of iNOS and SOCS3 (Figure 3a,b). The model has been submitted to the BioModel database with ID MODEL2005140001. Later, both the models were subjected to Flux analysis, followed by Sensitivity and Principal component analysis (PCA) (Supplementary Material S2).In this case, the PCA approach was laid down to identify multivariate relationship between IL6 signaling events and also link them to in vitro cellular phenotypes. We adopted the gold standard method of PCA and mathematical modeling approaches to more accurately differentiate between disease progressions states. These multivariate analyses led us to churn out the major reactions that add to disease progression at cellular level (Table 1), followed by the Ordinary differential equation of (ODEs) of major biochemical reactions (Supplementary Material S2D).

(1) $$\begin{array}{l} \frac{d\left(\left[TLR2/6-IL6\right]\cdot Vmembrane\right)}{dt}\\ =+Vmembrane\;\cdot \left(8e-09\;\cdot \left[TLR2\right]\cdot \left[\frac{TLR6}{1}\right]\cdot Vmembrane\;\cdot \left[LPG\right]\cdot Vmembrane\right)-(0.23\cdot [“TLR2/6\\ -LPG”]\cdot Vmembrane) \end{array}$$

(2) $$\frac{d\left(\left[MyD88\right]\cdot Vcytosol\right)}{dt}\;=-V\;cytosol\;\left(\frac{15\;\cdot \left[MyD88\right]}{1+\left[MyD88\right]\cdot VCYTOSOL}\right)+\left(0.23\cdot \left[“TLR2/6-LPG”\right]\cdot Vmembrane\right)$$

(3) $$\frac{d\left(\left[STAT3.P\right]\cdot VCYTOSOL\right)}{dt}\;=-VCYTOSOL\;\cdot \left(0.05\;\cdot \left[STAT3.P\right]\right)+VCYTOSOL\;\cdot \left(\frac{15\;\cdot \left[STAT3\left\{CYTOSOL\right\}\right]}{1+\left[STAT3\left\{CYTOSOL\right\}\right]\cdot VCYTOSOL}\right)\;+VCYTOSOL\left(\frac{1000\;\cdot \left[STAT3\left\{CYTOSOL\right\}\right]}{\;5000+\left[STAT3\left\{CYTOSOL\right\}\right]\cdot VCYTOSOL}\right)$$

(4) $$\frac{d\left(\left[SOCS3\left\{CYTOSOL\right\}\right]\cdot VCYTOSOL\right)}{dt}=+\left(0.5\;\cdot \left[SOCS3\left\{NUCLEUS\right\}\right]\cdot VNUCLEUS\right)$$

(5) $$\frac{d\left(\left[SOCS1\left\{CYTOSOL\right\}\right]\cdot VCYTOSOL\right)}{dt}=+\left(0.3\cdot \left[SOCS1\left\{NUCLEUS\right\}\right]\cdot VNUCLEUS\right)$$

### 2.2. Systems Study Reveals Phosphorylated STAT1 and STAT3 as Cross Talk

**Figure 4:**
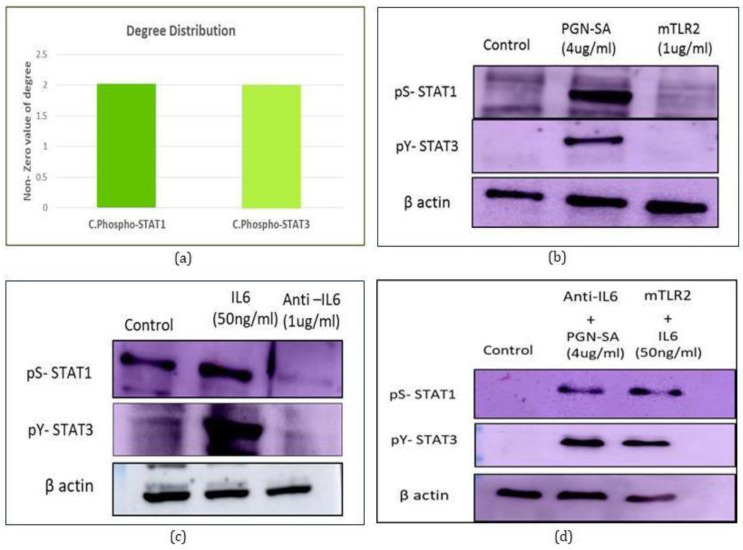
Identification of cross talks between TLR2/TLR6-IL6 signaling pathways (**a**) Degree distribution of cross talks identified through network analysis (**b**,**c**) Expression levels of phosphorylated STAT1 (S727) and STAT3 (Y705) during activation and inhibition of their respective pathway (**d**) Western blotting was used to check expression levels of phosphorylated STAT1 (S727) and STAT3 (Y705) during activation of one pathway & inhibition of another pathway, and vice versa. For all western blot experiments, an equal amount of protein was resolved and blotted for b-actin to ensure equal loading. The experiments were performed thrice. Results from one representative experiment are shown.

At the intracellular level, the model captures a variety of signaling events, the most important being the signal transduction networks emanating from receptors and engaging downstream in cross-talk. With respect to our previous observation [11] and now in refined models as well, cytoplasmic phosphorylated STAT1 and STAT3 are found to act as cross talk points between TLR2/TLR6-IL6 signaling pathway (Figure 4a), later validated through western blotting. We observed that, in both cases, the activation of either of the two pathways (TLR2 or IL6 signaling pathway) resulted in the phosphorylation of STAT1 and STAT3, and further, the phosphorylation is inhibited with the addition of their respective pathway inhibitors (Figure 4b,c). Subsequently, the addition of activator of TLR2 and inhibitor of IL6 signaling pathway resulted in phosphorylation of STAT1 & STAT3. Similar results have been obtained when treated with activator of IL6 and inhibitor of TLR2 signaling pathway, which lead to the conclusion that phosphorylated STAT1 and STAT3 acts as a cross talk point between IL6 and TLR2 signaling pathway. 

### 2.3. Multi Objective Optimization of Mathematical Model

The idea of performing multi-objective optimization of mathematical models is to elucidate how the network is evolvable with respect to changing environmental conditions. The evolved network could be a better platform to generate any kind of therapeutics. *Leishmania* interferes with the IL6 signaling network by modulation of SOCS1:SOCS3 ratio. SOCS1 is responsible for anti-inflammatory behavior and SOCS3 corresponds to pro-inflammatory behavior. Once the rewiring of the network is completed, the network should evolve towards pro inflammatory phenotype. Since obtaining evolvability of a system is a multi-optimization problem, we opted for Multi-objective genetic algorithm (MOGA) and defined objective function as:

function y = SOCS (x)

y(1)= −((exp(1* x(1)) + abs(1* x(2)) + 2* x(3) + abs(2* x(4))))^3^; Pro-inflammatory

y(2)= ((exp(2* x(1)) + abs(2* x(2)) + 1* x(3) + abs(1* x(4)))^2^; Anti-inflammatory

end

**Figure 5:**
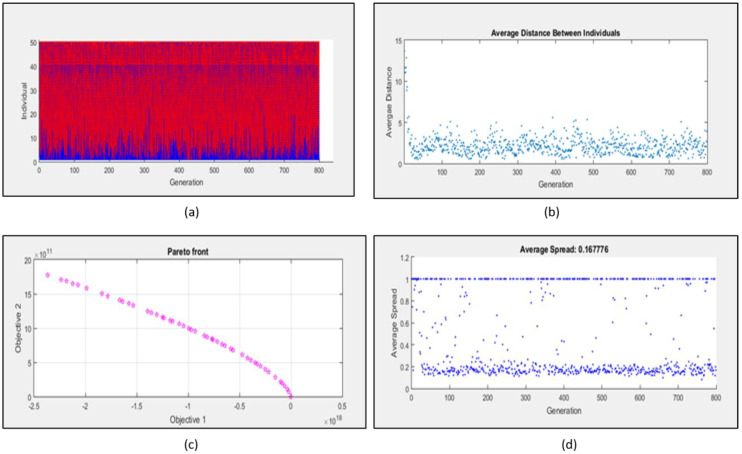
Multi objective genetic algorithm for optimization of SOCS1/SOCS3 ratio (**a**) Graph of elite population (**b**) Graph showing average distance between individuals indicating the decreased mutation rate (**c**) Pareto front between two objective function representing non-dominated solution (**d**) Graph depicting the average spread measure of the solution.

The decision variables embedded in y(1) and y(2) objective functions are cytokines, whose expression levels would be measured in in vitro experiments. They are denoted as x1, x2, x3 and x4, representing the cytokines IL10, TGFβ, TNFα and IFNγ, respectively. It shows that x1 and x2 decision variables represent the anti-inflammatory cytokines for the fitness function of SOCS1 protein (y(1)). Similarly, x3 and x4 represent (pro-inflammatory) fitness function of SOCS3 protein. When a genetic algorithm is performed with defined objective functions, a graph of individual v/s generation is obtained showing elite population (Figure 5a), this signifies the importance of SOCS1:SOCS3 ratio, as a character of elite population. The average distance between the individuals is low throughout the run, indicating decreased mutation rate or conservedness of the ratio throughout many generations (Figure 5b). The Pareto front obtained for the opposing objective functions has more than 30 non-dominated solutions that are not discontinuous and the average spread measure for these solutions is 0.167776 (Figure 5c,d). The Pareto optimality obtained using genetic algorithm states that during the process of natural selection the ratio of SOCS1:SOCS3 obtained through mathematical modeling analysis (Figure 5c) together with the anti and pro-inflammatory cytokine is selected (conserved) and passed onto next generation as an elite character. By targeting this conserved ratio, the designed therapeutics would be more effective for generations turning the macrophage polarization towards M1 phenotype. 

### 2.4. SOCS1 as a Target for Therapeutic Intervention

By performing the process of model reduction, there are various other reactions which have been filtered out in both the models (Table 1). One of the major reactions with high flux during analysis was the formation of active cytoplasmic SOCS1 protein (SOCS1 {NUCLEUS} -> SOCS1 {CYTOSOL}) in DSM. Cytoplasmic SOCS1 was among the major nodes identified through principal component analysis and also showed high sensitivity score (Supplementary Material S2A). Thus, cytoplasmic SOCS1 is selected as a target for further therapeutic intervention.

### 2.5. Peptide Design, Docking and MD Simulation of Selected Complex

**Figure 6:**
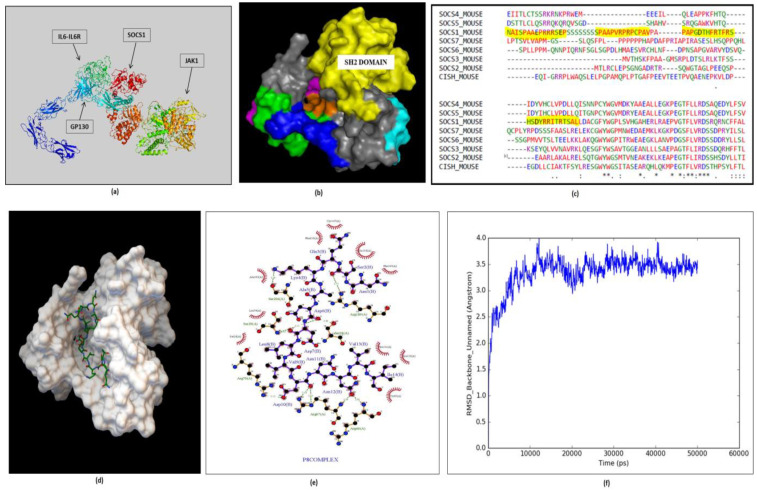
Peptide designed against SOCS1 protein: (**a**) Protein–protein docking of IL6-IL6R-gp130 complex with JAK1 and SOCS1 to identify interfacial residue during interaction. (BioLuminate Package, Schrödinger Release 2017-3 Suites). (**b**,**c**) Multiple sequence alignment identified non-conserved regions near the SH2 domain of SOCS1 protein as a target for peptide design. (**d**) The complex shows that peptide 8 has been docked at desired region near SH2 domain that may inhibit the interaction of SOCS1 and gp130 (Autodock v1.5.6) (**e**) Interaction plot of SOCS1 and Peptide8 amino acid residues (Ligplot+). (**f**) Molecular dynamics simulation of SOCS1-peptide 8 complexes have been performed for 50 ns depicting RMSD plot with stabilized fluctuations, indicating the stability of the SOCS1-Peptide 8 complex.

Suppressor of cytokine signaling (SOCS) is a negative feedback regulator of JAK-STAT signaling pathway; it binds to gp130 and inhibits the phosphorylation of STATs, thereby inhibiting JAK-STAT pathway.We designed a small peptide library of 15 peptides based on Machine Learning (Supplementary Material S3A), assumption was on the non-conserved region in SOCS1 protein (Figure 6a–c). Peptide 8 (NSQKADDLVDNNVI) was selected on the basis of number of interacting residues as well as low energy complex forming ability (Figure 6d,e) (Supplementary Material S3B). The SOCS1-Peptide8 complex was then subjected to 50 ns MD simulation. The RMSD plot shows that the complex got stabilized post 20 ns and the complex achieved its minimum energy state conformation (Figure 6f) (Supplementary Material S4A–S4B). 

### 2.6. Systems Driven Synthetic Biological Circuit Design

**Figure 7:**
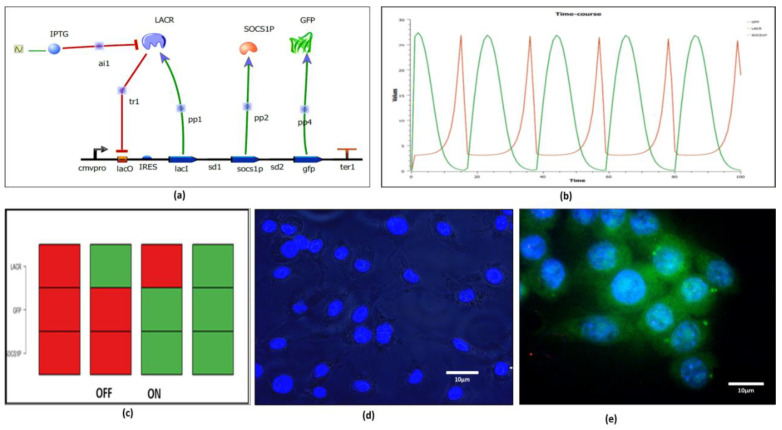
Synthetic circuit Design: (**a**) Modular arrangements of biological parts to receive an output in Tinker Cell. (**b**) After simulating the system, the output is received in the form of oscillatory behavior of LACR, Peptide8 and GFP. (**c**) Attractor states of synthetic circuits show its ON and OFF stage. Plasmid was transfected and expressed in the RAW264.7 cell line, through IPTG induction, followed by DAPI staining. (**d**) OFF stage of the system in vitro (no IPTG induction). (**e**) ON stage of the system in vitro (with IPTG induction).

Mathematical modeling in systems biology helps mimic the natural designs already existing for synthetic/genetic circuits, which is anticipated through creation of single/multi network as the case may be based on the knowledge of the underlying mechanism or on instinct of the researcher. For addressing the present issue, we have designed a mammalian tunable synthetic repressilator. The designed system contains *LacI*, *peptide 8*, *gfp* genes arranged in modular fashion under the influence of CMV promoter (Supplementary Material S5). IL6 synthetic gene circuit is constructed by assembling all necessary parts and pools. Parts are well defined DNA sequences with assigned roles in transcription or translation. Pools are the theoretical settings where free molecules of signal carriers are deposited. The transcription units of parts interact by exchanging molecules of signal carriers such as transcription factors, etc., and organize themselves into higher modules. Thus, when dealing with the circuit dynamics, parts and pools are modeled in an unassisted fashion through the exchange of fluxes of signal carriers in consonance with the mass action kinetics. These fluxes furthermore determine input/output in the genetic circuit and influence circuit performance. Promoters as signal carriers serve as parts that bind to RNA polymerase and do acquire the value of free signal carrier molecules concentration into their analogous pools. The system is auto negative regulatory in nature due to the presence of the Lac repressor gene (LacI) and its function is inhibited by IPTG (Figure 7a,b). In the absence of IPTG, the system remains turn off (OFF STAGE) signifies no production of GFP (green fluorescent protein) and peptide 8, whereas, in presence of IPTG the system is in ON STAGE representing the production of GFP and peptide 8. Both the stages have been confirmed through the BoolNet package and transfected successfully in the macrophage derived cell line (Figure 7c–e) (Supplementary Material S6 & S7A). The simulations have been performed for 100 min with the graph representing oscillatory behavior confirming the auto negative regulatory nature of the designed system. The wiring graph obtained shows that the Lac repressor is the center/master for regulation of the whole system (Supplementary Material S7B). Using its time series data, convergence of statistical variables have been obtained, signifying modularity as well as orthogonality (Supplementary Material S7C). The null-cline point obtained through an ODE solver states that the system has one stable state at a given time point; the system will either be in an ON state or in OFF stage (Supplementary Material S7D). The results further imply that the system has a tendency to follow the same trajectory even in presence of external perturbations, known as canalization. Computational method to study circuit canalization is very similar to the sensitivity analysis. 

### 2.7. In Vitro Validation

#### 2.7.1. Cytokine Profiling

To determine the efficacy of the designed synthetic circuit, cytokine profiling for various groups were performed using Taqman chemistry. Miltefosine is taken as positive control and the study was divided into five groups namely Control (C), Infection(I), Empty Vector (EV), Transfection + Infection (CTI), Transfection + Infection + Miltefosine (CTIM).

Control (C): Untreated RAW 264.7 cell line.

Infection (I): RAW 264.7 cell line infected with stationary phase promastigotes.

Empty Vector (EV): RAW 264.7 cell line transfected with empty vector without synthetic circuit.

Transfection + Infection (CTI): RAW 264.7 cell line transfected with designed synthetic circuit followed by induction with IPTG and then infection. 

Transfection + Infection + Miltefosine (CTIM): RAW 264.7 cell line transfected with the designed synthetic circuit, induced with IPTG followed by infection and then further treated with miltefosine. 

**Figure 8:**
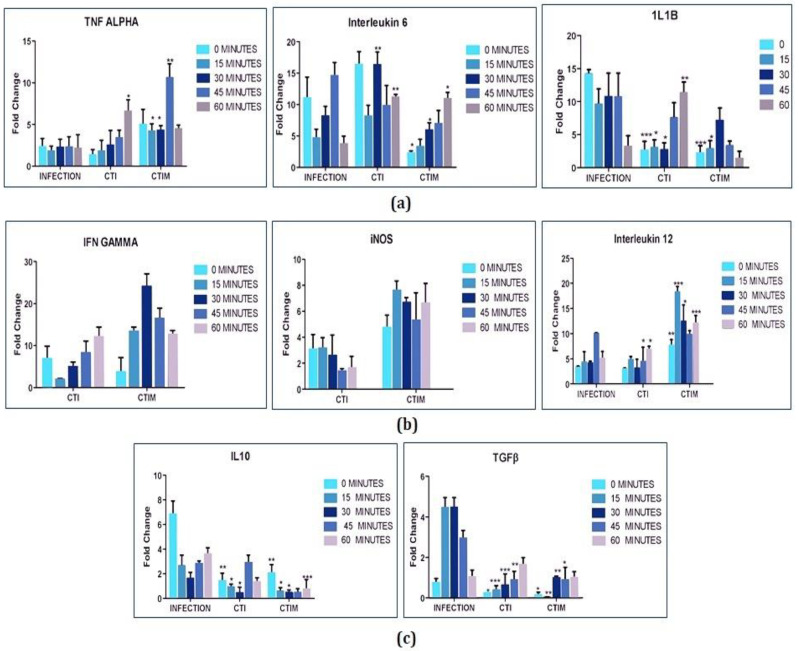
Cytokine profiling of synthetic circuit: Macrophages were first transfected with synthetic circuit using, Polyethylenimine (3:1 ratio PEI:DNA) and then induced with 1 mM IPTG for 48 h followed by infection with stationary phase promastigotes in 1:10 macrophage to parasite ratio for 24 h. Un-internalized parasites were washed off and samples were collected at different time points. (**a**) Cytokines released during initial interaction. (**b**) Cytokines associated with M1 phenotype. The levels of IFNγ and iNOS have not been observed in the Infection group therefore, bars are not shown. (**c**) Cytokines associated with M2 phenotype. C: Control, I: Infection with *L. major*, EV: Transfection of Cells with empty vector. CTI: Transfection of cell with synthetic circuit and then infection. CTIM: Transfection of cell with synthetic circuit and then infection followed by Miltefosine treatment. The experiments were performed thrice. The error bars represents mean ± SD with *p* value * *p* < 0.05, ** *p* < 0.01, *** *p* < 0.001.

During the initial interaction, cytokines having pro-inflammatory behavior are found to increase with the introduction and expression of the synthetic circuit. The expression was further increased with the treatment of Miltefosine. If we observe the cytokine profiling of the infectious state, TNFα shows a constant 2–3 fold change which symbolizes its role in the parasite clearance during initial infection (Figure 8a) but the fold change of IL12 (2–5 fold change) is low as compared to the fold change of IL10 (7–8 fold change). This predominantly shows the negative regulation of IL10 over IL12 which turns the polarization of macrophages towards M2 phenotype (Figure 8a,c). Additionally, there was no expression of IFNγ and iNOS which is due to the constant increase in the fold change of TGFbeta (0–45 min post infection), depicts that TGFβ is a strong anti-inflammatory cytokine which suppresses the expression of IFNγ and iNOS (Figure 8b,c). IL1β has both pro and anti-inflammatory behavior, the increase in fold change of IL1β is in synchrony with IL10 and TGFβ which shows its predominant anti-inflammatory action in establishing infection during early stage (Figure 8a,c). There was no expression of IL4 observed post one hour of infection. With the introduction of the designed synthetic circuit in infected cells, there is rapid increase of TNFα which has been observed with nearly 10 fold change (60 min post infection) and which further increases with Miltefosine treatment (Figure 8a). This proves that the designed circuit promotes the levels of TNFα in micro-environment establishing anti-leishmanial response during the early stage of the infection. Furthermore, sharp falls in fold change of IL10 and TGFβ have been observed. The fold change levels of IL10 have reached to 1–3 times from 7–8 times, whereas the fold change of TGFβ has been dropped from 4–5 times to 1–2 times (Figure 8c) representing the negative regulation of TNFalpha over IL10 and TGFbeta and thus shifting the polarization towards M1. Although there is not much increase in fold change of IL12, if we observe minutely, reciprocity in regulation has been observed between IL10 and IL12 at 45–60 min post infection (Figure 8a,c). The major increase in fold change of IFNγ and inducible nitric oxide synthase (iNOS) shows that introduction of the designed synthetic circuit is tilting the macrophage phenotype towards classical activation, resulting in killing of the parasite inside the macrophages (Figure 8b). From the cytokine profiling of various groups, we can conclude that Peptide8 inhibits the binding of SOCS1 with gp130, restores the phosphorylation of STAT1 and activation of JAK-STAT1 signaling pathway, thereby formation of pro-inflammatory cytokine during infection. 

#### 2.7.2. Nitrite Estimation

**Figure 9:**
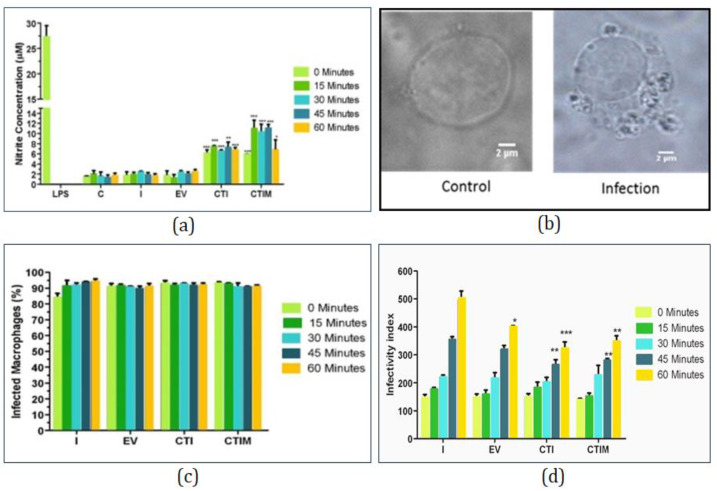
In vitro validation of designed synthetic circuit: (**a**) Nitrite estimation for various groups post one hour of infection in comparison with LPS treatment. (**b**) Microscopic image (100×) of control and infected RAW264.7 macrophage. (**c**) The percentage of macrophages infected with *L. major* parasite. (**d**) Estimation of parasite burden in terms of infectivity index in various infected and transfected groups. The experiments were performed thrice. One way ANOVA (with Tukey’s correction) have been used to perform statistical analysis of Infectivity index and student *t*-test have been used to analyze Nitric oxide estimation The error bars represents mean ± SD with *p* value * *p* < 0.05, ** *p* < 0.01, *** *p* < 0.001.

The potential of the designed synthetic circuit was further validated by quantifying nitrite in the system, which is indicative of macrophage polarization towards M1 phenotype. The study is further divided into five groups as mentioned above and Lipopolysaccharide (LPS) is taken as positive control. Production of nitrite among control, infection and empty vectors are found to be similar but when a designed synthetic circuit is induced in infected cells, nitrite production is increased which shows that the IL6 synthetic biological circuit is shifting macrophage polarization towards M1 phenotype. With the treatment of miltefosine, nitrate production is further increased (Figure 9a). 

#### 2.7.3. Parasite Load Assay

After nitrite estimation, parasite burden has been represented and estimated in various groups (Figure 9b–d). No changes in parasite burden have been observed post 30 min of infection either in transfected or non-transfected systems. During 45–60 min post infection, the fold change in iNOS and IFNγ (Figure 8b) have been increased together with high nitrite production (Figure 9a) resulting in significant decrease in parasite load in transfected system (CTI and CTIM) (Figure 9c,d).

## 3. Discussion

The robustness of a biological network is determined by the regulation of the network at two levels, i.e., transcriptional level and translational level. During infection, the parasite modulates the immune signaling at both the levels resulting in establishment of the disease. In recent times, pathology of a disease is studied at systems level using advanced technologies such as genome wide DNA sequencing, Chip-seq, and Quantitative mass spectroscopy (including iTRAQ and SILAC), which showcase complete transcriptomics and proteomics profile of the diseased state. The data provide a wide variety of information regarding activation or inactivation of specific transcription factors, kinases, ubiquitinase or any specific pathway during diseased conditions. Using the advanced dataset together with the computational tool, a system immunologist can create a spatiotemporal model for disease pathogenesis which captures the host-immune dynamics and helps in identification of the key components that are crucial for a dynamical cellular response during infection. The model could serve as a base for designing therapeutics to rewire the immune signaling. 

Our aim in this paper is to illustrate the application of mathematical modeling of IL6 signaling network towards better understanding the immune response to *L. major*. To this end, we developed a spatiotemporal model tracking cytokine networks in *L. major* infection by solving the ordinary differential equations using MATLAB. This model serves as a template for the design of machine learning derived peptides based IL6 synthetic circuit. The model, combined with experimental data, captured the host-immune dynamics of parasitic infection and helped identify key components that are crucial for explaining individual variability of different cytokines for a dynamical cellular response. Identification of key components in these complex networks and linking these multivariate interactions to events at different physiological scales, for example, tissue level behavior that directly contributes to diseased states, is the crux in systems immunology. Interleukin 6 is one of the major targets of therapeutic design in various autoimmune disease and infectious diseases. Tocilizumab, a humanized anti-IL6R monoclonal Ab of the IgG1 class is widely used to treat rheumatoid arthritis [26], Castleman’s Disease [27],amyloid A amyloidosis [28], etc. IL6 is considered to be an important biomarker for novel SARS-CoV2 infection [29,30] and anti-IL6 treatment are currently under trials [31,32]. 

One of the prime important aspects of a synthetic biology approach is that one can rationally design tailor-made molecular devices to target a specific function in the cell that results in less off-target effects. The impact of this approach has been visible in the present work, where we are selectively targeting the anti-inflammatory effect of IL6 during *L. major* infection by selecting the specific molecule of downstream signaling (SOCS1) instead of inhibiting the entire IL6 signaling. During the process of model refinement in this paper, we have identified the ratio of SOCS1:SOCS3 as 3:2 for establishment of infection which is further exploited as a target for designing therapeutics. The elevated levels of SOCS1 protein (60,000 molecules/cell) have been mathematically quantified and found to inhibit the signaling of pro-inflammatory cytokines such as IL12, IFNγ and TNFα. Further, this inhibition resulted in increased production of anti-inflammatory cytokines (around 2–7 fold changes have been found as compared with control samples). Model analysis at various levels such as flux, sensitivity and principal component analysis represented the key reactions governing the dynamics of diseased state and SOCS1 playing a crucial role in the same. To add to this, structural analysis of these proteins helped identify specific regions responsible for its inhibitory action. The region is then targeted by designing a set of peptides (NSQKADDLVDNNVI) against it. The in silico design and analysis of the SOCS1- peptide complex enables us to test the efficiency in in vitro condition. In order to make the delivery of the peptide more specific and less expensive, we opt for synthetic biology approach, wherein an inducible gene regulatory circuit delivers the designed peptide at specific location (in present work it is in cytoplasm where there is production of SOCS1 protein). Here, the circuit design is precise as well as simple to avoid unnecessary complications during its transfection or else, similar to a Rube Goldberg machine, the designed circuit may look exciting in silico but would rarely yield informative results in wet lab conditions. The confirmatory analysis of the designed therapeutic shows a remarkable up regulation in pro-inflammatory cytokines such as TNFα, iNOS, IFNγ, IL12 and consequently down regulation of anti-inflammatory cytokines IL10 and TGFβ is noted. We observed that the infected cells transfected with the synthetic circuit showed higher expression of pro-inflammatory cytokines such as iNOS and IFNγ with 2–7 fold change as compared to the infection group. Moreover, the transfected & infected cell shows a significant reduction in the expression level of anti-inflammatory cytokines such as IL10 and TGFβ (3–5 fold reduction) as compared to the infected group. Cytokine profiling confirms that the macrophage polarization is shifting towards M1 phenotype characterized by observing decreased parasite load in transfected & infected groups (30 min post one hour of infection). The in vitro validation of the designed synthetic circuit confirms its anti-leishmanial activity and has proven the potential of systems derived synthetic gene regulatory circuits in therapeutics. 

In view of the above-mentioned, one of the major aspects of this current concept is that, by using a system driven synthetic immunology approach, we have actually targeted the host system (which contributes to disease progression) rather than the parasite itself counteracting the issue of resistance development. The inducible nature of the synthetic circuit ensures the control over the designed product and its action over the host system at cellular level. Robustness/homeostasis of the synthetic circuit is quantifiable in terms of its measure to the fluorescence level, which in a way governs the stability. Interestingly, it might become difficult to engineer in vivo, which might counteract synchronization of cell immunologic response over a population. Additionally, a scrupulous calibration of kinetics is obligatory in order to guarantee accepted signal transmission along the IL6 cascade. A negative feedback loop identified through mathematical modeling of IL6 signaling network facilitates the response time of IL6 synthetic circuit leading to the counterbalance of protein concentration. As discussed, the major limitation of the systems comes when the therapy is taken at complex biological level such as tissue/organ or entire organism. At a higher biological level, the system design is achieved with respect to the complexity of the biochemical network vis-a-vis combining the present design of the synthetic circuit with CRISPR-Cas9 system for its better performance in in vivo system [33]. Nonetheless, it will make the system much more bulky, which eventually affects its transfection efficiency. We have performed our studies with IL6 cytokine in cell culture where we cannot rule out the fact that these concentrations might be higher than found in a more tightly regulated in vivo environment. The reason for this is associated with the pathological process of the disease. In addition, disease activity and disease severity have a strong correlation which suggests that if we are able to possibly find a raised concentration of a particular cytokine, then that is indicative of its pathogenic relationship to the disease, or its value as a disease marker. 

Advancement in systems and synthetic immunology has shown great potential in designing future strategies to overcome the limitation of classical biomedical therapies. The wide range of applications includes pathogen characterization [34,35], screening assays [36,37], diagnostics [38,39,40], drug production [41] and vaccination [42] leading to the emergence of new and less expensive therapeutics. 

The major highlight we want to bring in is that whatever may be the plausibility of the conclusions per se, there is a definite certainty that detailed comprehension of the cytokine network has enhanced our knowledge of the Leishmania disease process. From a computational and mathematical point of view, the cytokine network has information captured in terms of the state and extent of the leishmania disease with the continuing responses and a yield that influences a generation of selected effector cells along with the coordination of new responses. Fine tuning of the biological response may pose a hindrance at a higher level. Nevertheless, it is evident that synthetic biology approach is still among the prominent and most appropriate choice for designing new therapeutics regime because of its specificity, cost efficiency and less off target effects. In a nutshell, we have reconstructed the TLR2-TLR6-IL6 signaling to decipher the host- parasite interaction during the early stage of leishmanial infection. The model serves as a basis for designing an IL6 gene regulatory circuit which activates the JAK-STAT1 signaling pathway and thus rewires the IL6 signaling to shift the paradigm to M1 phenotype. 

## 4. Materials and Methods

### 4.1. In Silico

#### 4.1.1. Reconstruction and Analysis of IL6 Mathematical Model 

Data fitting was performed with respect to the ratio of SOCS1/SOCS3, obtained from wet lab experimentation (Western Blotting) followed by optimization wherein respective parametric changes have been performed to fine tune the models. The entire data sets were simulated with a deterministic approach using 15s ODE solver followed by sensitivity analysis which quantifies the dependency of model trajectories upon variation in introduced parameters. Further, quantification of prediction uncertainties has been performed. Model construction, refinement and analysis have been performed in Simbiology Matlab Tool box (v7.11.1.866) and Copasi (v4.19). 

#### 4.1.2. Multi Objective Optimization and Evolvability 

The macrophage phenotype network have been optimized by defining two objective functions, f(1): SOCS1 associated with anti-inflammatory response, f(2): SOCS3 associated with pro-inflammatory response. We have used a Multi-objective Genetic algorithm (MOGA) to optimize the ratio of SOCS1:SOCS3 [43]. The optimization was performed in MATLABs’ Optimization toolbox (7.11.1.866) (MathWorks Inc., Portola Valley, CA, USA) using the function solver “gamultiobj”.

#### 4.1.3. Target Identification and Protein-Protein Docking

The refined models have further been analyzed and reduced which crucified Suppressor of Cytokine Signaling 1 (SOCS1) as the target. 3D structure prediction have been done for various proteins of IL6 signaling complex (mSOCS1, mgp130, mIL6R and mJAK) using ab initio modeling techniques (Robetta) and homology modeling (Modeller 9.18) apart from mIL6 (PDB ID: 2L3Y). SOCS1 protein was docked with IL6 signaling complex to identify interfacial residues involved in interaction (Figure 6a). Most of the residues belong to SH2 domain of SOCS1 and henceforth non-conserved region around SH2 domain is targeted for peptide designing using Dead End Elimination algorithm. The non-conserved regions were identified through multiple sequence alignment of all the SOCS1 proteins of the mouse (MultAlign) (Figure 6c). 

#### 4.1.4. Peptide Design, Docking and MD Simulations

Peptide library was designed using deterministic search method (Dead End Elimination) and secondary structure was obtained through PEPstrMOD [44], followed by docking against SOCS1 through Autodock Vina (v1.5.6) [45] and interaction studies through Ligplot [46]. Selected peptide-protein complex was further subjected to molecular dynamic simulations for 50 ns to study its stability in physiological condition. MD simulation was performed using DESMOND 3.2 (D.E. Shaw Research) from Maestro 8.2 [47], in explicit TIP3P water model using orthorhombic box with a default 10 nm cutoff PBC (periodic boundary condition) for a time period of 50 ns with the time steps of 2 fs. The RMSD, RMSF and the trajectories were analyzed using simulation event analysis in Desmond 3.2. 

#### 4.1.5. Synthetic Circuit Design and Quasipotential Landscape 

Designing and simulation of synthetic circuits was performed in Tinker cell (v1.2.693) [48]. Modularity and orthogonality of the circuit was confirmed through BoolNet [49]. Time series data for 100 min were generated through Gene Regulatory Network Inference using Time Series (GRENITS) (v 1.24.0) [50,51]. Various parts of synthetic circuit were obtained from Registry of Standard Biological Parts (iGEM) (Table 2) and assembled in Snapgene (v3.2.1)with pcDNA 3.1 (vector) (Supplementary Material S5 and S7B), followed by its procurement in the form of plasmid from Gene art Thermofisher Scientific. Stability of the synthetic circuit was achieved by obtaining its nullcline state through Berkeley Madonna (Version 9.1.3), followed by obtaining quasipotential landscape through equation Vq = −((LacR) + (peptide:8))*DT derived from Waddington’s epigenetic landscape [52]. 

### 4.2. In Vitro

Cell culture and parasites—The pathogenic promastigotes form of *Leishmania major* strain (MHOM/Su73/5ASKH) were maintained in Roswell Park Memorial Institute (RPMI) 1640 with 20% fetal bovine serum (Sigma, Kansas City, MO, United States) and 50 µg/mL penicillin. The parasites were passed regularly through BALB/c mice by injecting stationary phase promastigotes in the subcutaneous region of the footpad. After 4 weeks of infection, the mice were sacrificed and parasites were obtained from the lymph node of the infected footpad. This procedure was performed in order to maintain the virulence of the parasite [53]. The murine macrophage cell line RAW264.7 was maintained at 37 °C with 5% CO_2_ in Dulbecco’s Modified Eagle Medium (DMEM) with 10% fetal bovine serum and penicillin (100 μg/mL). 

#### 4.2.1. Reagents, Antibodies, Probes and Constructs

All other chemicals were from Sigma-Aldrich, unless indicated otherwise. Antibodies for Western blotting such as anti-IL6 (be006) from Biocell, anti-phospho STAT3 (S2690), from Sigma and mouse IL6 (#5210), anti-phospho STAT1 (#9177), anti- SOCS1 (#2923) and anti-SOCS3 (#3950) were obtained from Cell Signaling Technology (CST). Taqman Chemistry was used to perform and quantify cytokine expression levels in samples. Mouse specific taqman probes (4331182) and Mastermix(4304437) were obtained from Thermofisher Scientific (Supplementary Material S8). Peptidoglycanfrom *Staphylococcus aureus* (PGN-SA) (#tlrl-pgns2) and Mab-mTLR2 (#mab-mtlr2) from InvivoGEN were procured. Designed synthetic circuit was procured in the form of plasmid from GeneArt, Thermofisher Scientific.

#### 4.2.2. Macrophage and Parasite Infection

For in vitro experimentation, RAW 264.7 cell line was infected with stationary phase promastigotes in 1:10 macrophage/parasite ratio for 24 h, followed by thorough washing of un-internalized parasite thrice with PBS and incubating the infected cells in DMEM with 10% FBS for 0, 15, 30,45, 60 min post infection.

#### 4.2.3. Transfection of Macrophages

Macrophages were transfected with a designed synthetic construct (plasmid form) using Polyethylenimine transfection reagent in a 3:1 ratio of PEI to DNA (*w*/*w*). The transfected cells were induced by 1 mM IPTG (Isopropyl β-d-1-thiogalactopyranoside) for 48 h followed by infection. Transfected cells were visualized for GFP expression on the EVOS FL fluorescence microscope.

#### 4.2.4. mRNA Isolation, RT PCR and Real Time PCR

For cytokine profiling, after washing the uninternalized parasite, cells were lysed and total RNA was isolated at 0 min, 15 min, 30 min, 45 min and 60 min post-infection. The total RNA was isolated using TRI Reagent as per the manufacturer’s instructions. The cDNA synthesis was done using 2 μg of total RNA through high Capacity cDNA kit (Invitrogen) as per the manufacturer’s instructions.

Q-PCR was performed on StepOnePlus Real-Time PCR System (Thermo Scientific, Waltham, MA, USA). For each reaction, 5 μL Taqman Master mix (Invitrogen, Waltham, MA, USA), 1 μg cDNA as Template, 0.5 μg Taqman probes (Supplementary Material S8) was taken, and the reactions were performed on thin-wall 0.1 mL fast 96 well plate (Applied Biosystems, Waltham, MA, USA) for a total of 10 μL reaction mix. Relative quantitation was done using the comparative threshold (ΔΔCT) method. The mRNA expression levels of the target genes were normalized against those of β actin levels and expressed as relative fold change compared with untreated controls.

#### 4.2.5. Western Blotting

##### Cross Talk Validation

For activation, RAW 264.7 cell line was treated with TLR2 activator PGN-SA (Peptidoglycan of *Staphylococcus aureus*) for 24 h and for inhibition, culture was treated with 1 μg/mL of mTLR2 before activation. The activation of IL6 pathway has been performed by treating cells with mIL6 (50 ng/mL) for 24 h and for inhibition, anti-IL6 antibody (1 μg/mL) treatment was given for 1 h prior to IL6 treatment. 

##### SOCS1/SOCS3 Validation

The macrophage derived cell line RAW 264.7 cell lines were infected with a promastigote form of parasite in 1:10 ratio, followed by 24 h incubation and removal of undigested parasite. The culture was then treated with 50 ng/mL of mouse IL6 protein for another 1 h followed by sample collection.

For Western blotting, cells were treated with indicated reagents and lysed with lysis buffer (50 mM Tris [pH 7.5], 250 mM NaCl, 50 mM NaF, 10% glycerol, 5 mM EDTA, 0.5 mM Sodium orthovanadate, and 0.5% Triton X), and a protease inhibitor mixture, by incubation on ice for 20 min followed by centrifugation of lysates (15,000 rpm, at 4 °C for 20 min). Supernatants were quantified by Bicinchoninic acid kit (Thermofisher scientific). An equal amount of protein was loaded on SDS-PAGE, and resolved proteins were transferred to nitrocellulose membrane (Millipore, Billerica, MA, USA) and blocked with 3% Bovine serum albumin in TBST (20 mM Tris [pH 7.5], 150 mM NaCl, and 0.1% Tween 20). Membranes were incubated with primary Antibody (1:1000 dilution) at 4 °C overnight, followed by washing with TBST, and incubated with HRP-conjugated secondary Ab. Immuno-reactive bands were visualized with the Luminol reagent (Santa Cruz Biotechnology, Santa Cruz, CA, USA). Densitometric analysis of bands was performed using Image J software.

#### 4.2.6. Parasite Load Assay 

After specific treatment, macrophages were washed with 1X cold PBS followed by fixation in 4% paraformaldehyde (PFA), and permeabilization in 0.1% Triton X. Thereafter, the cells were stained with DAPI (1 µg/mL). Parasites per macrophage were calculated using EVOS FL fluorescence microscope and they were represented in terms of infectivity index (percentage of infected cells x number of parasites per infected cells). 

#### 4.2.7. Estimation of NO Production

Presence of Nitrite in culture media is an indicator of Nitric oxide production by cells (precisely macrophage polarization towards M1 phenotype). The cell culture supernatant in 150 µL volume is treated with 20 µL of Griess reagent (0.1% N-(1-naphthyl)ethylenediamine and 1% sulfanilic acid in equal volume; Thermofisher Scientific) to set a total volume of 300 µL per reaction and incubated for 10 min at room temperature followed by colorimetric estimation at 540 nm Lipopolysaccharide (LPS) is taken as positive control (1 μg/mL). 

#### 4.2.8. Animal Maintenance

Female BALB/c mice, 6–8 weeks old with 18–20 g weight, originally procured from The Jackson Laboratory (Bar Harbor, ME, USA) and maintained in the Experimental Animal Facility of National Centre for Cell Science (NCCS), Pune were taken. Animals were used according to the Institutional Animal Ethical Committee–approved animal use protocol (IAEC Project Number-IAEC/2016/B-269). Post experimentation procedures involved Euthanasia which had CO_2_ dose asphyxiation. Carcasses were bagged and stored in the freezer and handed over in color coded bags to Pune Municipal Corporation (PMC) authorized biomedical waste disposal agency at their regular collection intervals.

#### 4.2.9. Statistical Analysis

The in-vitro experiments were performed in triplicates. The error bars are represented as mean ± s.d. The statistical significance between the indicated experimental and control groups was deduced by using Student’s *t*-test and One way ANOVA (with Tukey’s correction).

## Figures and Tables

**Table 1:** Reaction that governs the disease progression at cellular level.

S.No.	Reactions	Flux (Molecules/s)	PCA Score
1.	TLR2/6-LPG -> MyD88	207.4748058	0.95
2.	JAK1 + STAT3{CYTOSOL} -> STAT3.P	666.8136585	0.73
3.	SOCS1{NUCLEUS} -> SOCS1{CYTOSOL}	15,045.06	0.9
4.	SOCS3{NUCLEUS} -> SOCS3{CYTOSOL}	4999.92	0.81

**Table 2:** Registry of standard biological parts with parts registry number (Sequences of the same enlisted in Supplementary Material S5).

S.No.	Biological Parts	Accession ID
1.	CMV promoter + RBS	BBa_I712004
2.	LacR	BBa_K731500
3.	GFP+ Terminator	BBa_K259006
4.	Spacer DNA	BBa_K1123011
5.	pcDNA 3.1 (Backbone)	BBa_K3030004

## Data Availability

The data that support the findings of this study are available in the Supplementary Materials.

## Supplementary Materials

The following are available online. S1: Concentration of components of mathematical model. S2A–S2D: S2A: Principal component analysis; S2B: Comparative flux analysis of both the models. S2C: Complete simulation graphs of both the models; S2D: ODEs of both the models (PDF); S3A–S3B: S3A: Peptide sequences and Docking Score; S3B: Characteristic of Peptide8 obtained from ExPASY ProtParam tool; S4A–S4B: S4A: RMSF Plot of SOCS1-P8 Complex; S4B: Physical parameters during 50 ns MD simulation; S5: DNA sequences of parts used in the synthetic circuit; S6: Insert Verification details (PDF); S7A–S7D: S7A: Plasmid map of the designed construct; S7B: Wiring of the circuit signifies the major regulatory axis as Lac Repressor gene. S7C: Convergence of statistical variables signifying the stability of the system. S7D: Nullcline form of the synthetic circuit with states depicting synthetic circuit reaching its equilibrium state. S8: Invitrogen Assay ID for RT PCR probes.

## Abbreviations

AIF	Anti-inflammatory factor
CMV	Cytomegalovirus
CL	Cutaneous leishmaniasis
CT	Comparative threshold
CTI	Control + Transfected + Infected
CTIM	Control + Transfected + Infected + Miltefosine
DAPI	4′,6-diamidino-2-phenylindole
DMEM	Dulbecco’s Modified Eagle Medium
DSM	Diseased State Model
EDTA	Ethylenediaminetetraacetic acid
EV	Empty Vector
GFP	Green Fluorescent Protein
HSM	Healthy State Model
IFNγ	Interferon gamma
IPTG	Isopropyl β-d-1-thiogalactopyranoside
iGEM	International Genetically Engineered Machine
iNOS	Inducible nitric oxide synthase
IL6	Interleukin 6
IL1β	Interleukin 1 beta
IL10	Interleukin 10
JAK/STAT	Janus kinase/signal transducer and activator of transcription
LACR	Lactose Repressor
LPS	Lipopolysaccharide
MOGA	Multi-objective genetic algorithm
ODE	Ordinary differential equation
PBC	Periodic boundary condition
PCA	Principal component analysis
PFA	Paraformaldehyde
PGN-SA	Peptidoglycan from *Staphylococcus aureus*
PEI	Polyethylenimine
RPMI	Roswell Park Memorial Institute
RMSD	Root-mean-square deviation
RMSF	Root mean square fluctuation
SH2	Src Homology 2
SOCS	Suppressor Of Cytokine Signaling
TIP3P	Transferable intermolecular potential with 3-point
TLR	Toll-like Receptors
