# Supplementary material for: Synthetic Perturbations in IL6 Biological Circuit Induces Dynamical Cellular Response
Source: Molecules. 2021 Dec 26;27(1):124. doi: 10.3390/molecules27010124 (PMC8746995; doi:10.3390/molecules27010124)
Supplement: Supplementary file 1 [file molecules-27-00124-s001.zip › molecules-1486900-supplementary.pdf]

SUPPORTIVE INFORMATION

S1: Concentration of components of mathematical model

COMPONENTS	HSM (molecules)	DSM (molecules)
TLR2	1000	1000
TLR6/1	1000	1000
IL6-IL6R	1000	1000
IL6R	1000	1000
Membrane IL6	1000	1000
LPG	100	1000
IL6-IL6R-GP130	2000	2000
IFN-g	1000	200
IFN-gR	1000	150
IFNg-IFNgR	1000	150
gp130	1000	1000
TLR2/6-LPG	900	900
IL10	100	1000
IL10R	100	1000
IL10-IL10R Complex	10000	10000
MyD88	800	800
IRAK1-IRAK4	700	700
TRAF6	600	600
TAK1-TAB1/2	500	500
IKKbeta	400	400
NFkB	300	300
Cytoplasm IL6	100	100
STAT3.P	0	10000
STAT1.P	80000	0
JAK1	20000	20000

2.STAT1	40000	0
2.STAT3	0	9000
JAK2/1	2000	2000
SOCS1	10000	10000
TLP2	200	200
NFkB	200	200
Nucleus IL6	250	250
Nucleus 2.STAT1	6000	0
Nucleus 2.STAT3	0	30000
Nucleus SOCS3	10000	10000
Nucleus STAT1	100	100
Nucleus STAT3	100	100
Nucleus SOCS1	4000	50000
iNOS	0	0
AIF	0	0

S2:

S2A: PRINCIPAL COMPONENT ANALYSIS

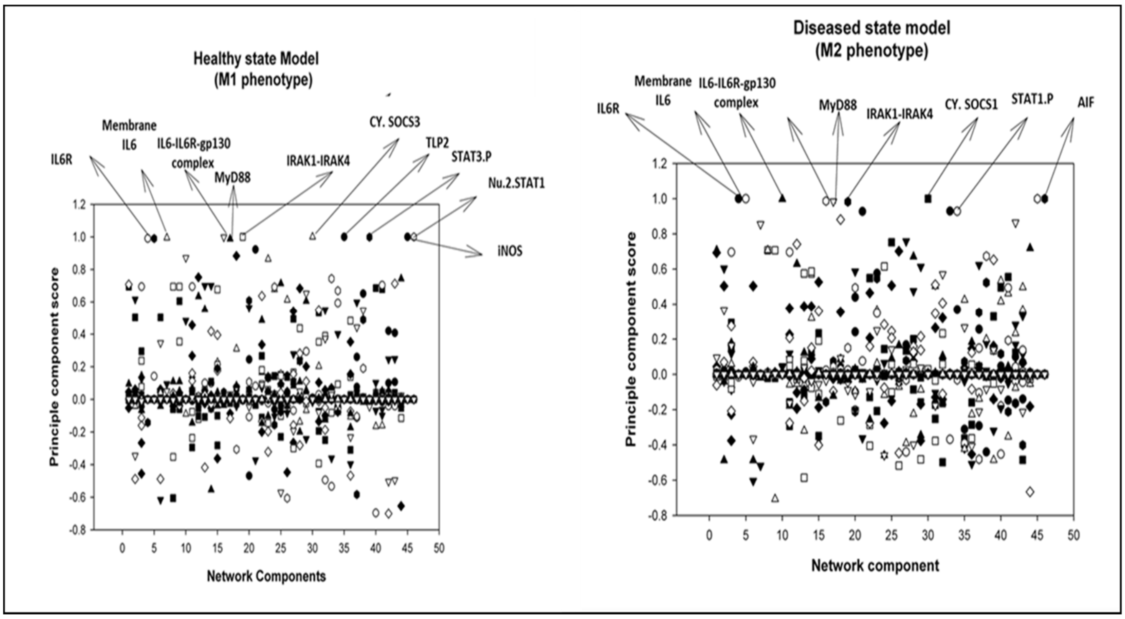

Fig S2A: Principle Component analysis of Diseased State and Healthy State Model

S2B: Comparative Flux analysis of both the models

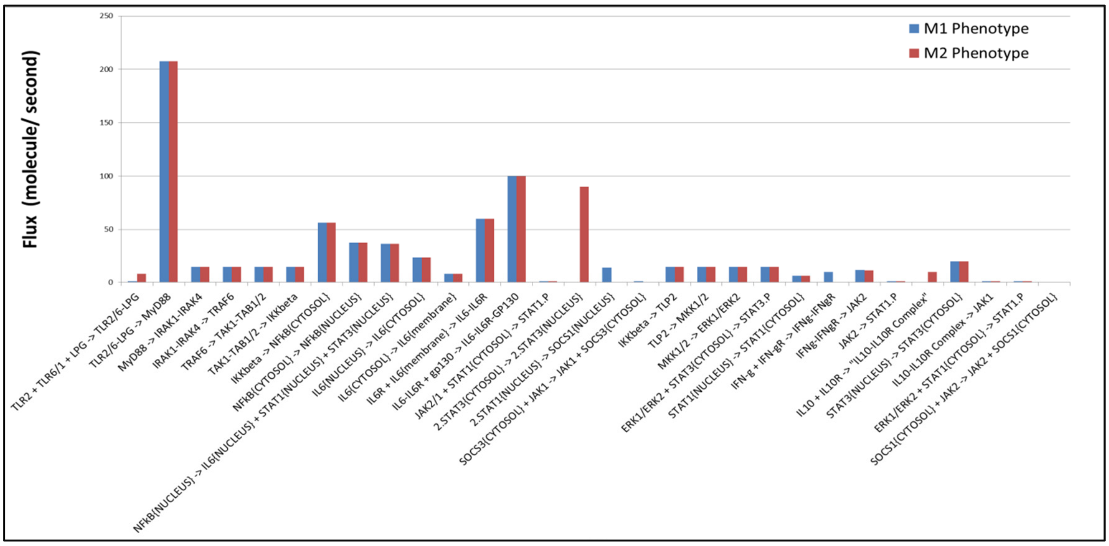

(a)

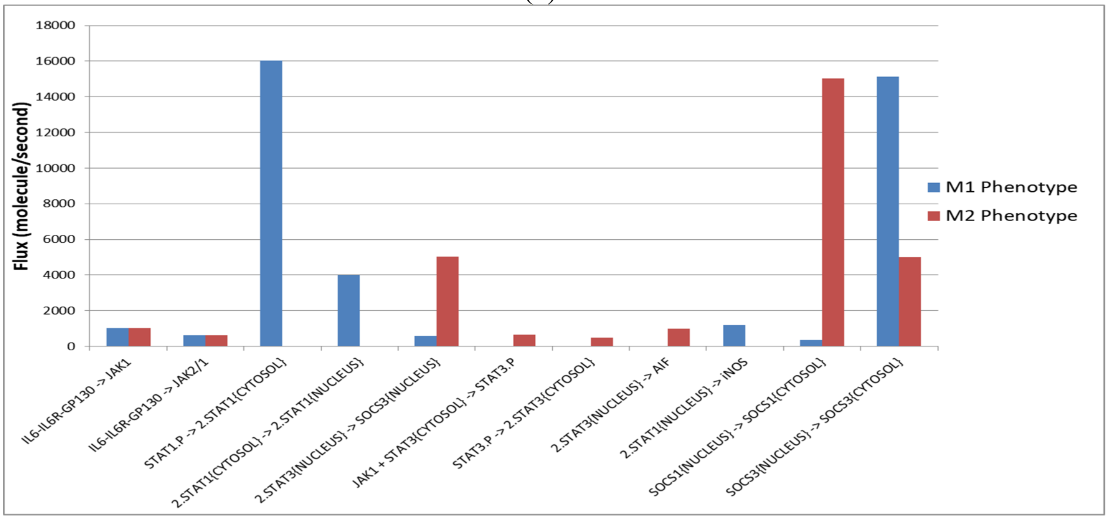

(b)

Fig. S2B: Flux analysis: (a) Comparative flux of Diseased state and Healthy state model (b) Reactions with greater flux in each model.

S2C: Complete Simulation graph of both models

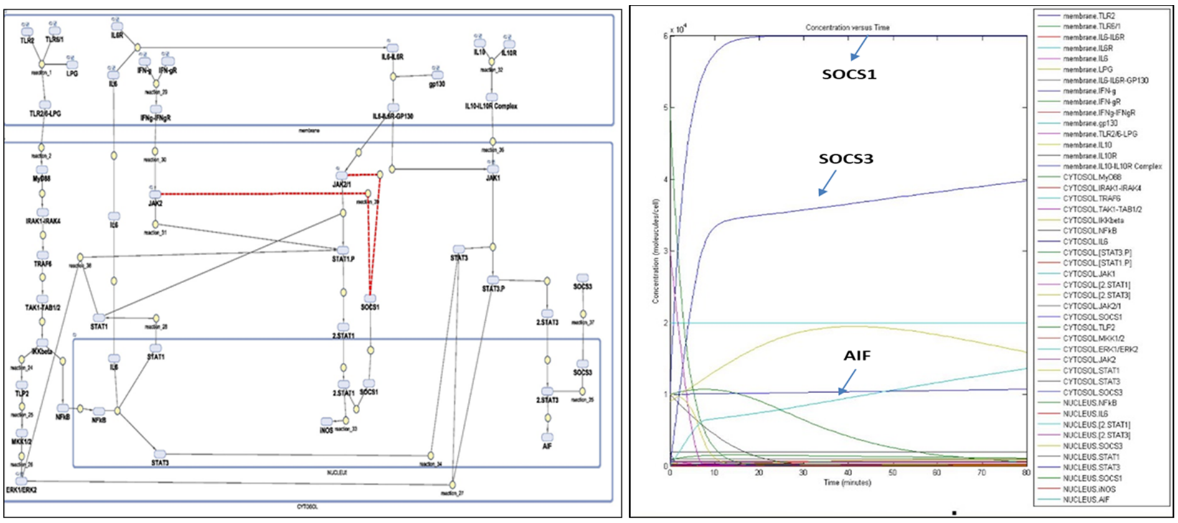

(a)

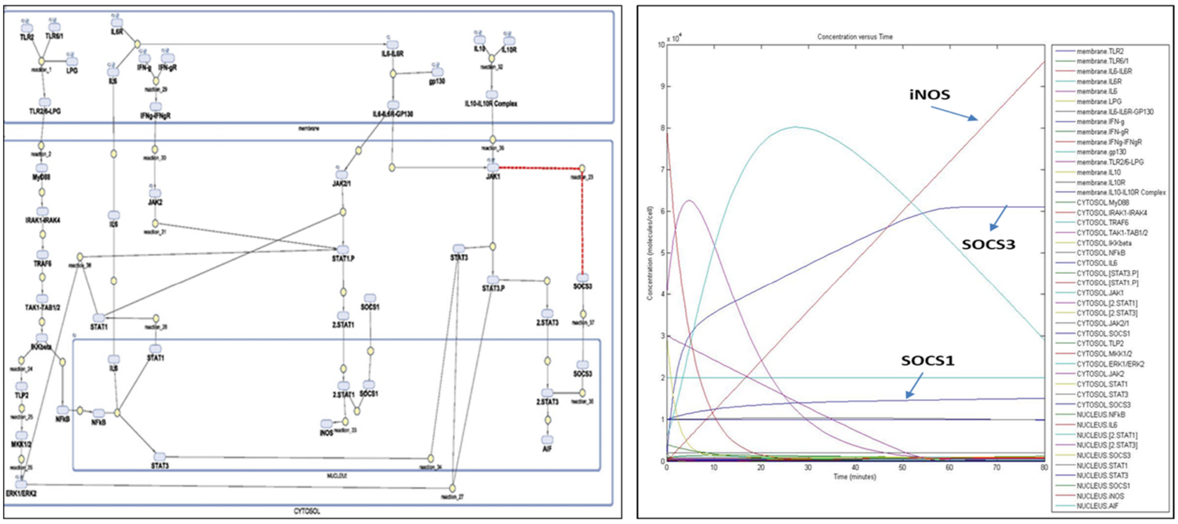

(b)

Fig. S2C: Mathematical Modeling: Diagrammatic representation and simulated graph of (a) M2 phenotype (b) M1 phenotype.

S3:

S3A: Peptide Sequences and its Docking score

S no.	Motif	Peptide Nomenclature	Peptide	Docking score
1.	APGDTHFRTFRSHS	P1	VHAKNSVDNADNTN	-6.2
		P2	AHVKNTAENVEQSQ	-6.0
		P3	VHAKNSVDNADN	-5.1
		P4	AHVKNTAENVEQ	-6.4
		P5	ADNTNCDCADDL	-6.4
2	GDTHFRTFRSHS	P6	AKNSADNVDNSQ	-5.5
		P7	VRQTVEQADQSN	-5.9
3	**SHSDYRRTTRTSAL**	**P8**	**NSQKADDLVDNNVI**	**-5.9**
		P9	QTNKVDELAQNNAI	-6.5
		P10	NSQKADDLVDNN	-6.0
		P11	QTNKVDELAQNN	-5.2
4.	RIVAAVGRENLA	P12	DLAVVAADKSIV	-5.7
		P13	ELVAAAVEKTLG	-5.9
5.	VAAVGRENLARI	P14	AVVAVDNSIVDL	-4.7
		P15	AVVAAENTIVEL	-6.4

S3B. Characteristic of Peptide8 obtained from ExPASY ProtParam tool:

Number of amino acids:	14
Molecular weight:	1544.64 Da
Theoretical pI:	3.93

Amino acid composition:

Ala (A) 1 7.1%	Arg (R) 0 0.0%
Asn (N) 3 21.4%	Asp (D) 3 21.4%
Cys (C) 0 0.0%	Gln (Q) 1 7.1%
Glu (E) 0 0.0%	Gly (G) 0 0.0%
His (H) 0 0.0%	Ile (I) 1 7.1%
Leu (L) 1 7.1%	Lys (K) 1 7.1%
Met (M) 0 0.0%	Phe (F) 0 0.0%
Pro (P) 0 0.0%	Ser (S) 1 7.1%
Thr (T) 0 0.0%	Trp (W) 0 0.0%
Tyr (Y) 0 0.0%	Val (V) 2 14.3%
Pyl (O) 0 0.0%	Sec (U) 0 0.0%

i. **Atomic composition:**

ATOM	ATOMIC WEIGHT
Carbon C	63
Hydrogen H	105
Nitrogen N	19
Oxygen O	26
Sulfur S	0

Formula: $C_{63}H_{105}N_{19}O_{26}$

Total number of atoms: 213

ii. **Instability index:**
The instability index (II) is computed to be 6.24. This classifies the protein as stable.

iii. **Aliphatic index:** 104.29

iv. **Grand average of hydropathicity (GRAVY)**: -0.764

S4:

S4A :RMSF plot of SOCS1-P8 complex:

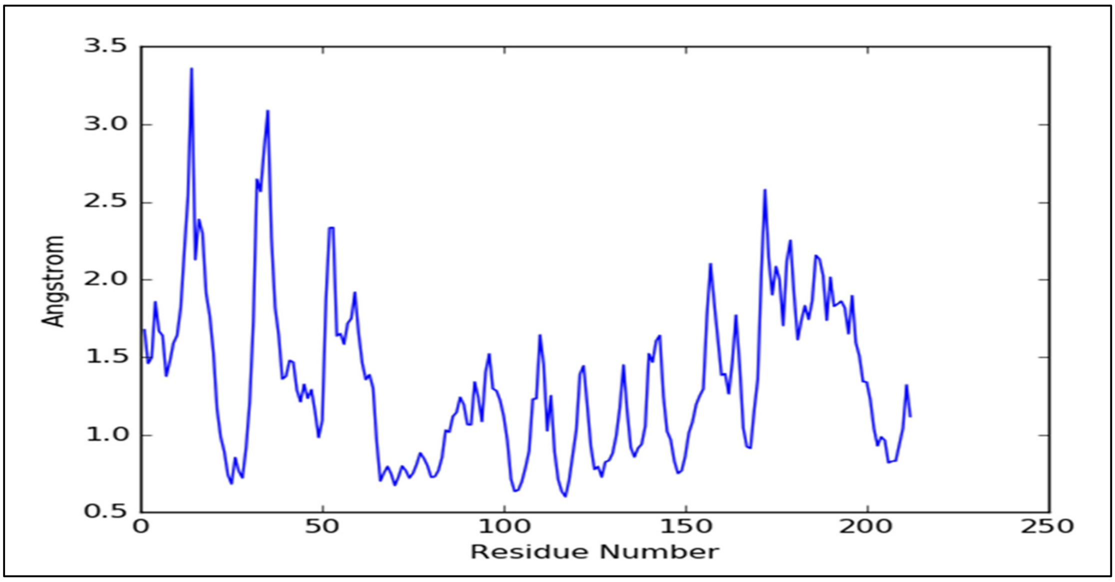

S4B: Physical parameters during 50nsMD simulation

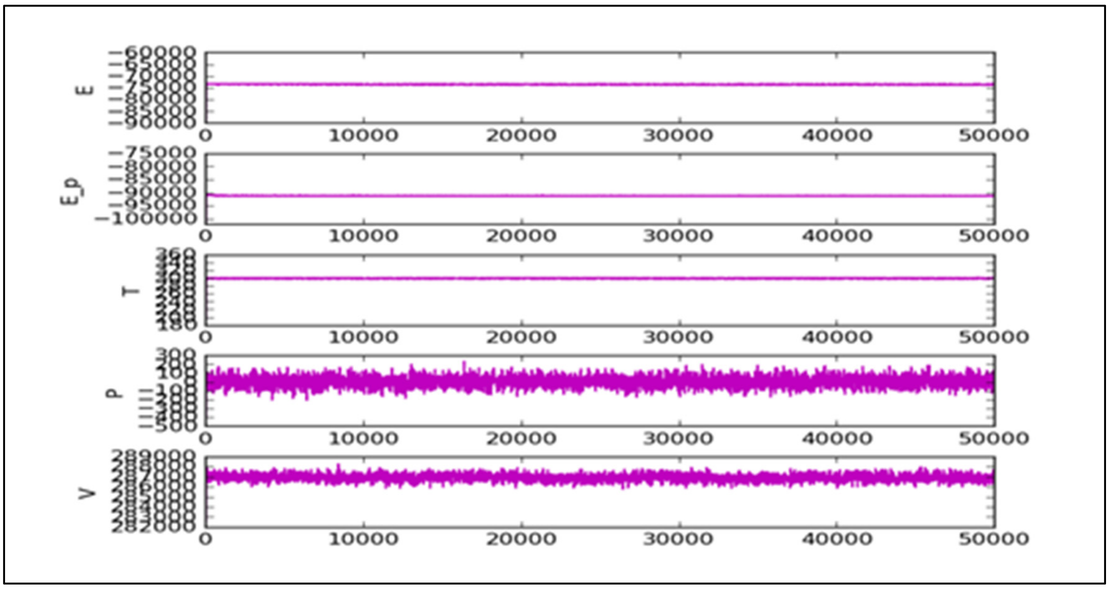

S5: DNA sequences of the parts used in the Synthetic Circuit

a. **CMV promoter + RBS (BBa_I712004) :**
CGATGTACGGGCCAGATATACGCGTTGACATTGATTATTGCCTAGTTATTAAT
AGTAATCAATTACGGGGTCATTAGTTCATAGCCCATATATGGAGTTCCGCGTT
ACATAACTTACGGTAAATGGCCGCCTGGCTGACCGCCCAACGACCCCCGCCC
ATTGACGTCAATAATGACGTATGTTCCCATAGTAACGCCAATAGGGACTTTCC
ATTGACGTCAATGGGTGGAGTATTTACGGTAAACTGCCCACTTGGCAGTACA

TCAAGTGTATCATATGCCAAGTACGCCCCCTATTGACGTCAATGACGGTAAAT
GGCCCGCCTGGCATTATGCCCAGTACATGACCTTATGGGACTTTCCTACTTGG
CAGTACATCTACGTATTAGTCATCGCTATTACCATGGTGATGCGGTTTTGGCA
GTACATCAATGGGCGTGGATAGCGGTTTGACTCACGGGGATTTCCAAGTCTC
CACCCCATTGACGTCAATGGGAGTTTGTTTTGGCACCAAAATCAACGGGACTT
TCCAAAATGTCGTAACAACTCCGCCCCATTGACGCAAATGGGCGGTAGGCGT
GTACGGTGGGAGGTCTATAAAGCAGAGCTCTCTGGCTAACTAGAGAACCCAC
TGCTTACTGGCTTATCG AAAT

b. **lacO2 operator (BBa_K731500):**
GTGAGCGGATAACAATTCCC

c. **LacI Repressor Protein (BBa_K731500):**
ATGAAACCAGTAACGTTATACGATGTCGCAGAGTATGCCGGTGTCTCTTATCA
GACCGTTTCCCGCGTGGTGAACCAGGCCAGCCACGTTTCTGCGAAAACGCGG
GAAAAAGTGGAAGCGGCGATGGCGGAGCTGAATTACATTCCCAACCGCGTG
GCACAACAACTGGCGGGCAAACAGTCGTTGCTGATTGGCGTTGCCACCTCCA
GTCTGGCCCTGCACGCGCCGTCGCAAATTGTCGCGGCGATTAAATCTCGCGC
CGATCAACTGGGTGCCAGCGTGGTGGTGTCGATGGTAGAACGAAGCGGCGTC
GAAGCCTGTAAAGCGGCGGTGCACAATCTTCTCGCGCAACGCGTCAGTGGGC
TGATCATTAACTATCCGCTGGATGACCAGGATGCCATTGCTGTGGAAGCTGCC
TGCACTAATGTTCCGGCGTTATTTCTTGATGTCTCTGACCAGACACCCATCAA
CAGTATTATTTTCTCCCATGAAGACGGTACGCGACTGGGCGTGGAGCATCTG
GTCGCATTGGGTCACCAGCAAATCGCGCTGTTAGCGGGCCCATTAAGTTCTGT
CTCGGCGCGTCTGCGTCTGGCTGGCTGGCATAAATATCTCACTCGCAATCAAA
TTCAGCCGATAGCGGAACGGGAAGGCGACTGGAGTGCCATGTCCGGTTTTCA
ACAAACCATGCAAATGCTGAATGAGGGCATCGTTCCCACTGCGATGCTGGTT
GCCAACGATCAGATGGCGCTGGGCGCAATGCGCGCCATTACCGAGTCCGGGC
TGCGCGTTGGTGCGGATATCTCGGTAGTGGGATACGACGATACCGAAGACAG
CTCATGTTATATCCCGCCGTTAACCACCATCAAACAGGATTTTCGCCTGCTGG
GGCAAACCAGCGTGGACCGCTTGCTGCAACTCTCTCAGGGCCAGGCGGTGAA
GGGCAATCAGCTGTTGCCCGTCTCACTGGTGAAAAGAAAAACCACCCTGGCG
CCCAATACGCAAACCGCCTCTCCCCGCGCGTTGGCCGATTCATTAATGCAGCT
GGCACGACAGGTTTCCCGACTGGAAAGCGGGCAGTAATAA

d. **Green Floroscence Protein+ Terminator (BBa_K259006):**
ATGCGTAAAGGAGAAGAACTTTTCACTGGAGTTGTCCCAATTCTTGTTGAATT
AGATGGTGATGTTAATGGGCACAAATTTTCTGTCAGTGGAGAGGGTGAAGGT

GATGCAACATACGGAAAACTTACCCTTAAATTTATTTGCACTACTGGAAAACT
ACCTGTTCCATGGCCAACACTTGTCACTACTTTCGGTTATGGTGTTCAATGCTT
TGCGAGATACCCAGATCATATGAAACAGCATGACTTTTTCAAGAGTGCCATG
CCCGAAGGTTATGTACAGGAAAGAACTATATTTTTCAAAGATGACGGGAACT
ACAAGACACGTGCTGAAGTCAAGTTTGAAGGTGATACCCTTGTTAATAGAAT
CGAGTTAAAAGGTATTGATTTTAAAGAAGATGGAAACATTCTTGGACACAAA
TTGGAATACAACTATAACTCACACAATGTATACATCATGGCAGACAAACAAA
AGAATGGAATCAAAGTTAACTTCAAAATTAGACACAACATTGAAGATGGAAG
CGTTCAACTAGCAGACCATTATCAACAAAATACTCCAATTGGCGATGGCCCG
TCCTTTTACCAGACAACCATTACCTGTCCACACAATCTGCCCTTTCGAAAGAT
CCCAACGAAAAGAGAGACCACATGGTCCTTCTTGAGTTTGTAACAGCTGCGG
GATTACACATGGCATGGATGAACTATACAAATAATAAGCTGCAAACGACGAA
AACTACGCTTTAGTAGCTTCCAGGCATCAAATAAAACGAAAGGCTCAGTCGA
AAGACTGGGCCTTTCGTTTTATCTGTTGTTTGTCGGTGAACGCTCTC

e. **Spacer (BBa_K1123011) :**
AGGTTCTGTTAAGTAACTGAACCCAATGTCGTTAGTGACGCTTACCTCTTAAG
AGGTCACTGACCTAACA

f. **PEPTIDE:8**
AACAGCCAGAAAGCGGATGATCTGGTGGATAACAACGTGATT

g. **IRES (Internal Ribosomal Entry site) (BBa_K813001):**
AGCCAAAATAATGATAACGAGAATAATATCAAGAATACCTTAGAACAACATC
GACAACAACAACAGGCATTTTCGGATATGAGTCACGTGGAGTATTCCAGAAT
TACAAAATTTTTCAAGAACAACCACTGGAGGGATATACCCTTTTCTCTCACA
GGTCTGCGCC

S6: Insert verification details (PDF file)

S7A: Plasmid map of Empty Vector and the Designed Construct:

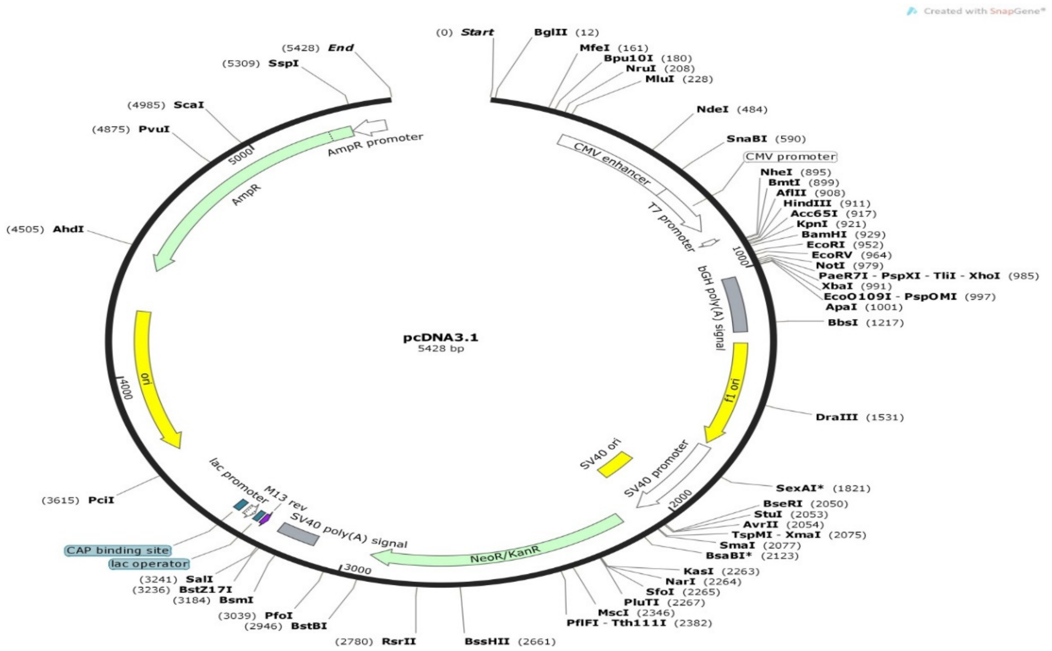
Created with SnapGene®
pcDNA3.1
5428 bp
(0) Start
BglII (12)
MfeI (161)
Bpu10I (180)
NruI (208)
MluI (228)
NdeI (484)
SnaBI (590)
CMV promoter
NheI (895)
BmtI (899)
AflII (908)
HindIII (911)
Acc65I (917)
KpnI (921)
BamHI (929)
EcoRI (952)
EcoRV (964)
NotI (979)
PaeR7I - PspXI - TliI - XhoI (985)
XbaI (991)
EcoO109I - PspOMI (997)
ApaI (1001)
BbsI (1217)
DraIII (1531)
SexAI* (1821)
BseRI (2050)
StuI (2053)
AvrII (2054)
TspMI - XmaI (2075)
SmaI (2077)
BsaBI* (2123)
KasI (2263)
NarI (2264)
SfoI (2265)
PluTI (2267)
MscI (2346)
PflFI - Tth111I (2382)
BssHII (2661)
(2780) RsrII
(2946) BstBI
(3039) PfoI
(3184) BsmI
(3236) BstZ17I
(3241) SalI
CAP binding site
lac operator
(3615) PciI
(4505) AhdI
(4875) PvuI
(4985) ScaI
(5309) SspI
(5428) End
CMV enhancer
T7 promoter
bGH poly(A) signal
f1 ori
SV40 ori
SV40 promoter
NeoR/KanR
SV40 poly(A) signal
M13 rev
lac promoter
ori
AmpR
AmpR promoter
1000
2000
3000
4000
5000

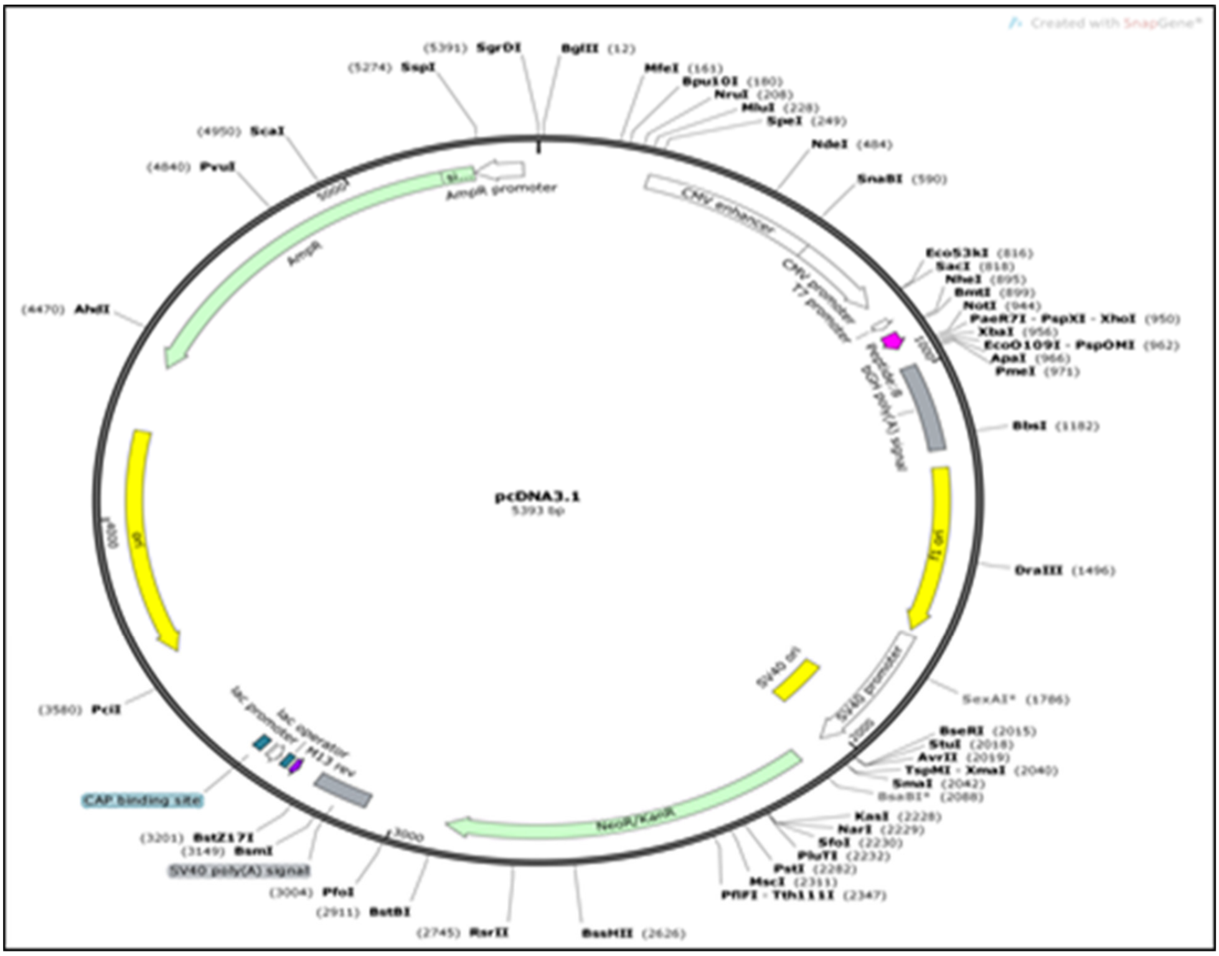
Created with SnapGene®
(5391) SgrDI
BglII (12)
(5274) SspI
MfeI (161)
Bpu10I (180)
NruI (208)
MluI (228)
SpeI (249)
NdeI (484)
SnaBI (590)
(4950) ScaI
(4840) PvuI
AmpR promoter
CMV enhancer
AmpR
CMV promoter
T7 promoter
Eco53kI (816)
SacI (818)
NheI (895)
BmtI (899)
NotI (944)
PaeR7I - PspXI - XhoI (950)
XbaI (956)
EcoO109I - PspOMI (962)
ApaI (966)
PmeI (971)
(4470) AhdI
BGH poly(A) signal
BbsI (1182)
pcDNA3.1
5393 bp
ori
f1 ori
DraIII (1496)
SV40 ori
SV40 promoter
SexAI* (1786)
(3580) PciI
lac operator
lac promoter
M13 rev
BseRI (2015)
StuI (2018)
AvrII (2019)
TspMI - XmaI (2040)
SmaI (2042)
BsaBI* (2088)
CAP binding site
NeoR/KanR
KasI (2228)
NarI (2229)
SfoI (2230)
PluTI (2232)
PstI (2282)
MscI (2311)
PflFI - Tth111I (2347)
(3201) BstZ17I
(3149) BsmI
SV40 poly(A) signal
(3004) PfoI
(2911) BstBI
(2745) RsrII
BssHII (2626)

S7B: Wiring of the circuit signifies the major regulatory axis as Lac Repressor gene.

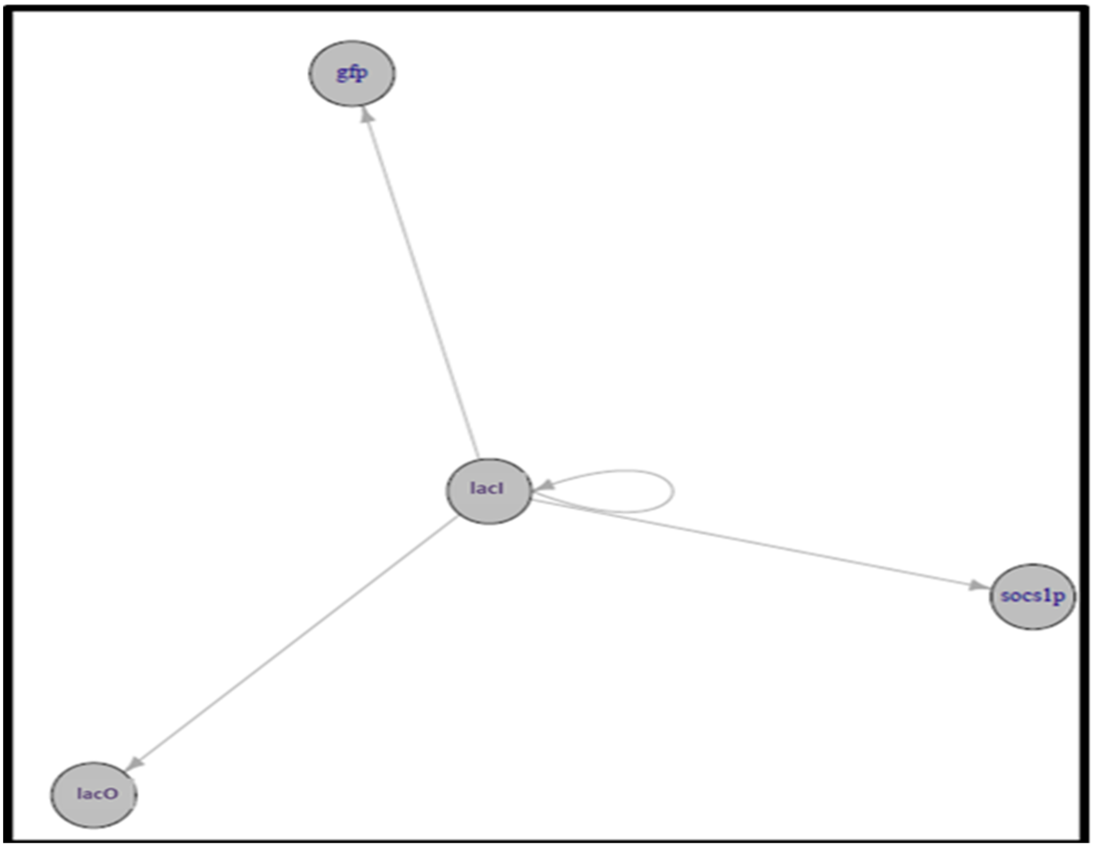

S7C: Convergence of Statistical Variables signifying the stability of the system.

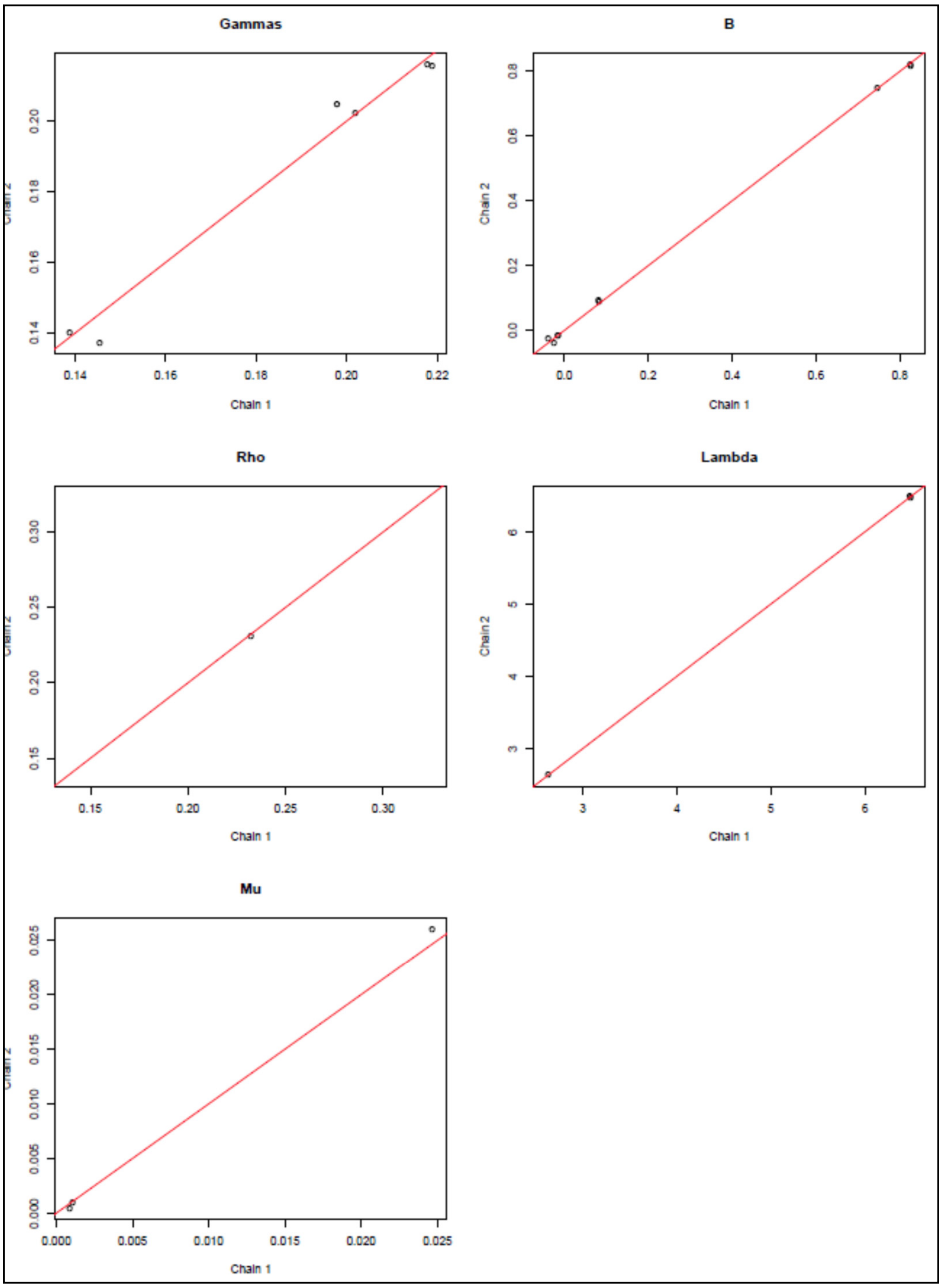

S7D: Nullcline form of the Synthetic Circuit with states depicting Synthetic Circuit reaching its equilibrium state.

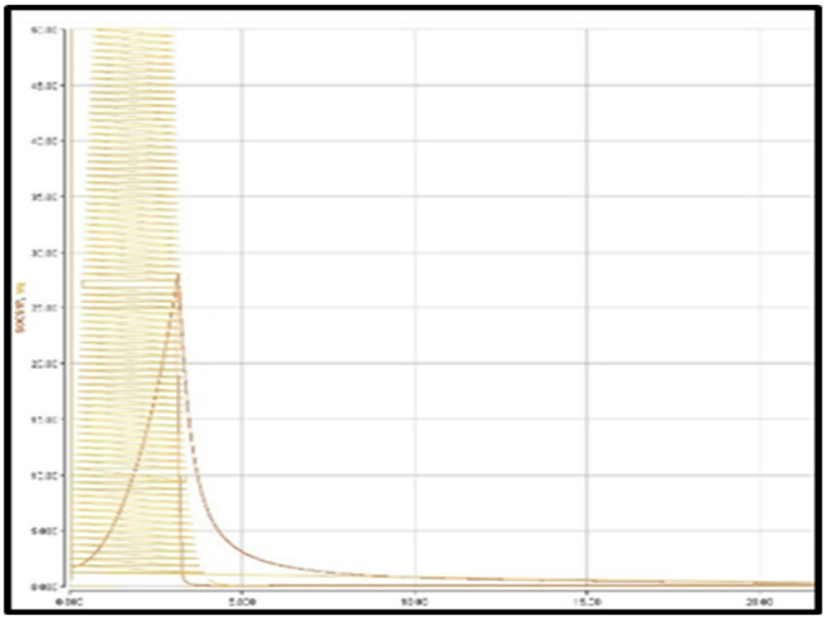

S8: Table of Invitrogen Assay ID for RT-PCR probes.

	Invitrogen® Assay ID	Gene
1.	Mm00445259_m1	IL4
2.	Mm00446190_m1	IL6
3.	Mm01288386_m1	IL10
4.	Mm00434165_m1	IL12α
5.	Mm00434228_m1	IFNγ
6.	Mm00443258_m1	TNFα
7.	Mm02619580_g1	ACTb
8.	Mm01321739_m1	TGFB
9.	Mm00434228_m1	IL1B
